# Nutrient regulation of the islet epigenome controls adaptive insulin secretion

**DOI:** 10.1172/JCI165208

**Published:** 2023-04-17

**Authors:** Matthew Wortham, Fenfen Liu, Austin R. Harrington, Johanna Y. Fleischman, Martina Wallace, Francesca Mulas, Medhavi Mallick, Nicholas K. Vinckier, Benjamin R. Cross, Joshua Chiou, Nisha A. Patel, Yinghui Sui, Carolyn McGrail, Yesl Jun, Gaowei Wang, Ulupi S. Jhala, Roland Schüle, Orian S. Shirihai, Mark O. Huising, Kyle J. Gaulton, Christian M. Metallo, Maike Sander

**Affiliations:** 1 Departments of Pediatrics and Cellular & Molecular Medicine, Pediatric Diabetes Research Center and; 2 Department of Bioengineering, UCSD, La Jolla, California, USA.; 3 Department of Urology, University of Freiburg Medical Center, Freiburg, Germany.; 4 Department of Molecular and Medical Pharmacology, David Geffen School of Medicine, UCLA, Los Angeles, California, USA.; 5 Department of Neurobiology, Physiology and Behavior, College of Biological Sciences, and Physiology and Membrane Biology, School of Medicine, UCD, Davis, California, USA.

**Keywords:** Endocrinology, Metabolism, Diabetes, Epigenetics, Islet cells

## Abstract

Adaptation of the islet β cell insulin-secretory response to changing insulin demand is critical for blood glucose homeostasis, yet the mechanisms underlying this adaptation are unknown. Here, we have shown that nutrient-stimulated histone acetylation plays a key role in adapting insulin secretion through regulation of genes involved in β cell nutrient sensing and metabolism. Nutrient regulation of the epigenome occurred at sites occupied by the chromatin-modifying enzyme lysine-specific demethylase 1 (Lsd1) in islets. β Cell–specific deletion of *Lsd1* led to insulin hypersecretion, aberrant expression of nutrient-response genes, and histone hyperacetylation. Islets from mice adapted to chronically increased insulin demand exhibited shared epigenetic and transcriptional changes. Moreover, we found that genetic variants associated with type 2 diabetes were enriched at LSD1-bound sites in human islets, suggesting that interpretation of nutrient signals is genetically determined and clinically relevant. Overall, these studies revealed that adaptive insulin secretion involves Lsd1-mediated coupling of nutrient state to regulation of the islet epigenome.

## Graphical abstract

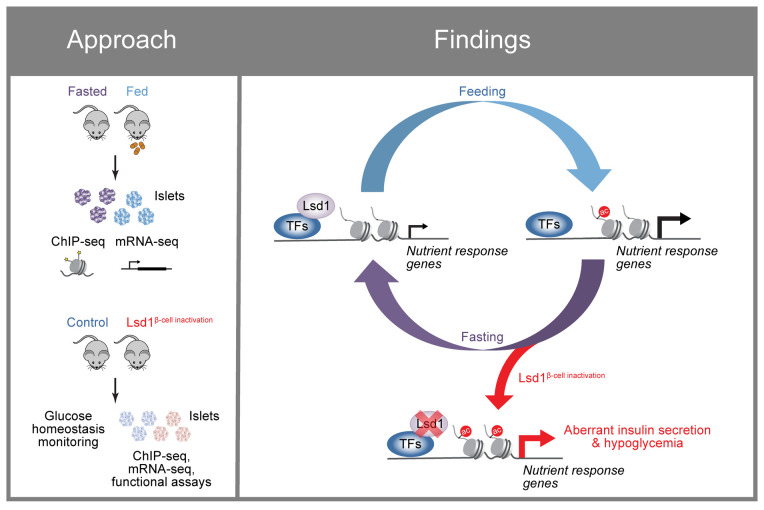

## Introduction

The ability to regulate nutrient metabolism in response to feeding and fasting is necessary for metabolic homeostasis. Nutrient utilization is acutely regulated by hormones and metabolites that change in response to feeding state (1). If an energy state persists, adaptive control mechanisms increasingly influence nutrient metabolism. Insulin produced by pancreatic β cells is the key stimulus for carbohydrate metabolism, and therefore it is critical that insulin secretion is adjusted commensurate with changes in energy state (1). The insulin-secretory response to feeding is primarily controlled by glucose and potentiated by intraislet glucagon (2) and incretin hormones such as Glp-1 through stimulation of cAMP production (3). The postprandial increase of serum glucose accelerates glucose metabolism within the β cell to initiate a signaling cascade involving ATP-stimulated plasma membrane depolarization, Ca^2+^ influx, and insulin vesicle exocytosis (4). Although much is known regarding how nutritional and hormonal signals acutely regulate the insulin-secretory response (3), it is unclear how feeding and fasting evoke sustained functional changes as β cells adapt to these nutrient states.

It has long been recognized that the insulin-secretory response is attenuated by prolonged fasting (5, 6) and sensitized in response to nutrient overload during adaptation to obesity (7, 8). During fasting, β cells rewire their metabolism to increase fatty acid oxidation at the expense of glucose metabolism, thereby reducing the ability of glucose to stimulate insulin secretion (6, 8, 9). This metabolic switch is in part transcriptionally driven through upregulation of Pparα (9). In the fed state, key nutritional signals that promote insulin secretion, such as glucose and Glp-1, also trigger changes in β cell gene expression over time courses relevant to feeding and fasting rhythms (10, 11), raising the possibility that these feeding-induced transcriptional changes contribute to adaptive enhancement of the insulin-secretory response. Nutrient signals mediate gene expression changes in part via signal-dependent transcription factors (TFs). For example, Glp-1 is known to mediate transcriptional changes through cAMP production and subsequent Creb activation, while glucose activates TFs through its metabolism both directly (in the case of Chrebp) and via β cell electrical stimulation, which activates nuclear factor of activated T cells (NFAT) via Ca^2+^ influx (12). Genome-wide analyses have implicated the glucose-activated TF Chrebp and the Glp-1/cAMP-activated TF Creb in broad transcriptional responses to these nutrient signals (13, 14).

Dynamics in nutrient state can additionally evoke changes to the epigenome through histone-modifying enzymes that utilize intermediary metabolites as cofactors or substrates or through indirect mechanisms via nutrient-responsive signaling pathways (15, 16). Gene regulation in response to glucose or Glp-1/cAMP has been associated with changes to histone acetylation in islets (14, 17), suggesting that chromatin modifications could contribute to nutrient-regulated transcription in β cells. However, it is unknown whether feeding is coupled to transcriptional or epigenomic regulation in β cells to mediate functional adaptation. One chromatin-modifying enzyme that modulates acetylation and methylation state in response to nutrient signals is the histone demethylase lysine-specific demethylase 1 (Lsd1) (18, 19). In adipose, Lsd1 functions as a signal-dependent chromatin modifier that adapts metabolism to changes in nutrient state (20, 21). Here, we show that feeding and fasting remodel the islet epigenome in an Lsd1-dependent manner to adapt the insulin-secretory response.

## Results

### Nutrient state regulates histone acetylation and transcription in pancreatic islets.

**Figure 1:**
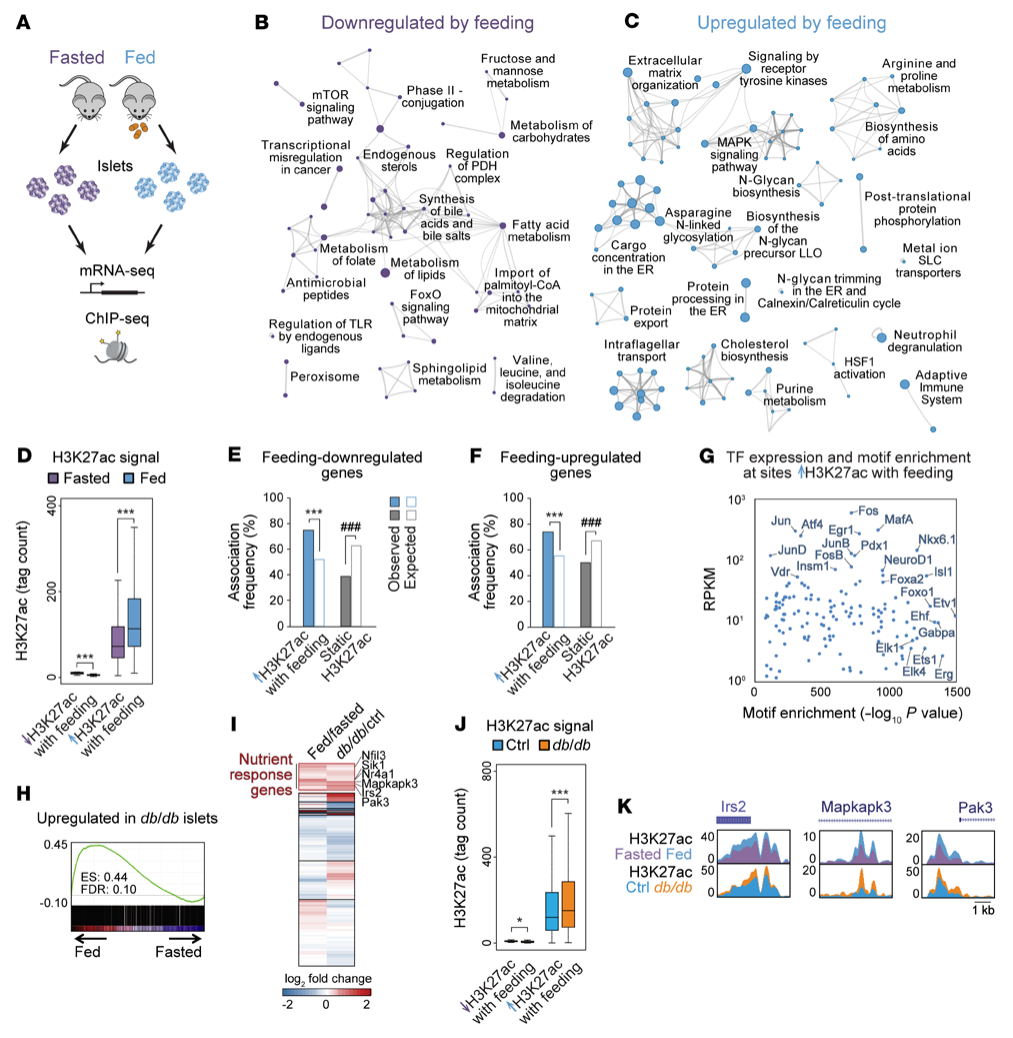
Nutrient state regulates histone acetylation and transcription in pancreatic islets. (**A**) Schematic of experiments performed. (**B** and **C**) Networks of genes downregulated (**B**) or upregulated (**C**) (*P* < 0.01 by Cuffdiff) by feeding relative to fasting, shown as clustered functional categories. *n* = 3. (**D**) H3K27ac ChIP-Seq signal at sites losing or gaining H3K27ac with feeding (*P* < 0.01 by DEseq2). *n* = 3. ***P < 0.001, Wilcoxon’s rank-sum test. (**E** and **F**) Association frequencies between TSSs of genes downregulated (**E**) or upregulated (**F**) by feeding with the indicated classes of H3K27ac peaks ± 10 kb. White bars indicate association frequencies expected by chance. ***P < 0.001 for enrichment; ^###^*P* < 0.001 for depletion; NS, not significant by permutation test. (**G**) TF motifs enriched at sites gaining H3K27ac with feeding relative to all other H3K27ac peaks plotted against mRNA levels of cognate TFs in islets from fed mice. (**H**) GSEA of genes upregulated in *db/db* compared with control (ctrl, *db/+*) islets (*P* < 0.01 by Cuffdiff; *n* = 3) against mRNA-Seq data from islets after feeding and fasting. (**I**) K-means clustering of log_2_ fold changes in mRNA levels in islets after feeding compared with fasting and in *db/db* compared with control islets. (**J**) ChIP-Seq signal for H3K27ac at the indicated classes of H3K27ac peaks in *db/db* and control islets. *n* = 2. ***P < 0.001, Wilcoxon’s rank-sum test. (**K**) H3K27ac ChIP-Seq genome browser tracks for the indicated genes. Box plot whiskers span data points within the interquartile range × 1.5.

To investigate adaptation of pancreatic islets to changes in nutrient state, we employed a time-restricted feeding paradigm (22) to reinforce natural rhythms of food intake in mice. Food availability was restricted to the 12-hour dark phase for 6 days, which did not result in weight loss (Supplemental Figure 1, A and B; supplemental material available online with this article; https://doi.org/10.1172/JCI165208DS1) and has previously been shown to not affect glucose tolerance (22). On the final day of entrainment after 12 hours of fasting, food was provided to one group for 4 hours and withheld from the fasted group, which resulted in differences in blood glucose levels (Supplemental Figure 1C). Glucose-stimulated insulin secretion (GSIS) by isolated islets was higher in the fed than in the fasted state (Supplemental Figure 1D), suggesting that even short-term changes in nutrient state trigger adaptive changes in the insulin-secretory response. To assess transcriptional changes associated with adaptation to feeding, we analyzed islets from fasted and fed mice by mRNA-Seq (Figure 1A and Supplemental Table 1), revealing 1,186 differentially expressed genes (*P* < 0.01; Supplemental Figure 1E and Supplemental Table 2A). Functional annotation of feeding-responsive genes identified regulation of metabolic (carbohydrate, lipid, and amino acid metabolism), nutrient-sensing (MAPK, mTOR, and FoxO), and other cell signaling (posttranslational protein phosphorylation) pathways (Figure 1, B and C) that could be involved in β cell adaptation to feeding.

Metabolic cues have been shown to effect transcriptional changes in part by regulating the epigenome (15, 16). In particular, acetylation of histone H3 Lys27 (H3K27ac), a histone modification associated with active promoters and enhancers (23) (“active chromatin” hereafter), is dynamically regulated in response to changes in nutrient state (15–17). To determine whether islet active chromatin is responsive to nutrient cues, we performed ChIP followed by sequencing (ChIP-Seq) for H3K27ac in islets from fasted and fed mice (Figure 1A and Supplemental Table 1). We found that 44% of H3K27ac peaks exhibited nutrient state–regulated changes in signal (*P* *<* 0.01 using DEseq2), of which nearly all (99.9%) gained acetylation with feeding (Figure 1D, Supplemental Figure 1, F and G, and Supplemental Table 3A). Feeding-induced H3K27ac deposition occurred independently of changes in monomethylation of H3 Lys4 (H3K4me1; Supplemental Figure 1H), a histone modification that cooccurs with H3K27ac in active chromatin (23). Sites gaining H3K27ac with feeding were enriched near both feeding-induced and feeding-repressed genes (Figure 1, E and F), suggesting effects of these chromatin changes on gene transcription. The paradoxical association between gains in H3K27ac with transcriptional downregulation is possibly indicative of a role for more distal regulatory elements or mechanisms independent of H3K27ac in controlling mRNA levels of these genes. Feeding-induced H3K27ac sites were enriched for motifs recognized by signal-dependent ETS and AP-1 family TFs (Figure 1G and Supplemental Table 4A), of which many were expressed in islets, and were also enriched for the motif of Foxo1 (Figure 1G), a fasting-activated TF with established functions in β cells (24). Together, these findings reveal that H3K27ac is dynamically regulated by feeding in islets and suggest signal-dependent regulation of chromatin at these sites.

During chronic overfeeding, augmented insulin secretion counteracts insulin resistance, thereby preventing hyperglycemia. To determine whether similar transcriptional and epigenetic changes occur in response to a sustained increase of nutrient intake, we analyzed islets from leptin receptor–deficient *db/db* mice at the onset of hyperglycemia, when *db/db* islets exhibit insulin hypersecretion indicative of an adaptive response (Supplemental Figure 1, I and J). Gene set enrichment analysis (GSEA) revealed significant overlap between genes upregulated in *db/db* islets and genes induced by feeding, including known regulators of nutrient signaling, such as *Irs2* (11), *Mapkapk3* (25), and *Pak3* (26) (Figure 1H, Supplemental Table 1, and Supplemental Table 2B). K-means clustering of islet gene expression changes during feeding and in *db/db* mice revealed a large cluster of 1,706 genes upregulated in both conditions (“nutrient-response genes” hereafter; Figure 1I). Supporting robustness of this nutrient-response program, we observed upregulation of this gene set in published islet mRNA-Seq data from mice chronically fed a high-fat diet (Supplemental Figure 1K). Analysis of chromatin state in *db/db* islets further revealed hyperacetylation of H3K27 and hypermethylation of H3K4 at active chromatin sites, most prominently at feeding-induced H3K27ac sites (Figure 1, J and K, Supplemental Figure 1, L–N, and Supplemental Table 1). Together, these findings indicate that chronic overfeeding augments nutrient-stimulated histone acetylation concomitant with increased histone methylation and recapitulates a disproportionate share of feeding-induced gene expression changes.

### The histone demethylase Lsd1 is recruited to feeding-regulated active chromatin during fasting.

**Figure 2:**
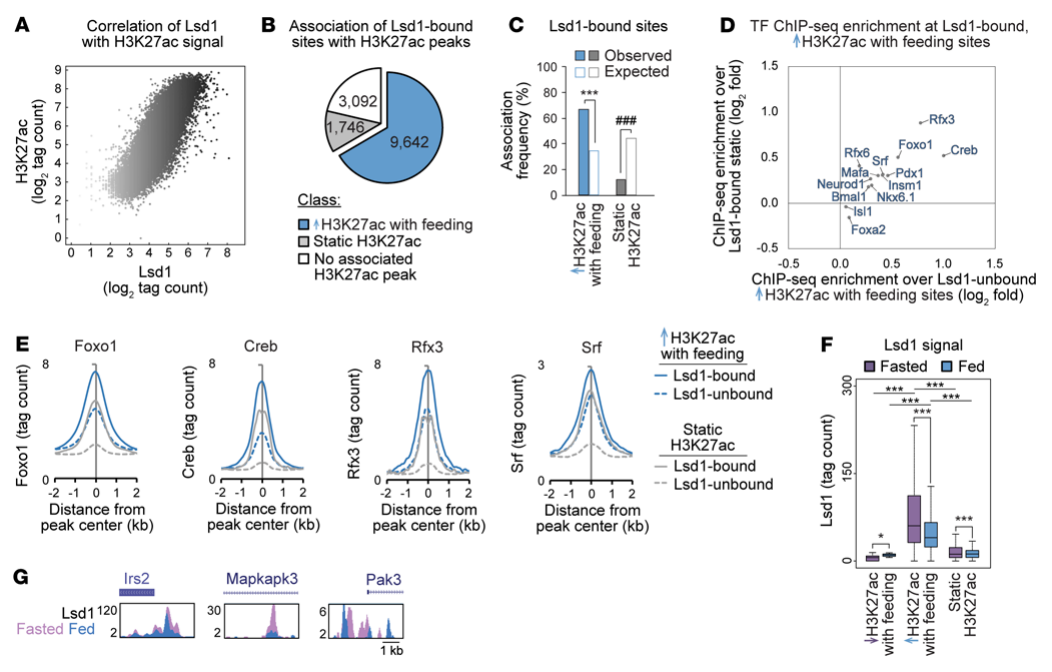
Lsd1 is recruited to feeding-regulated active chromatin at sites bound by nutrient-regulated TFs. (**A**) Scatterplot of Lsd1 and H3K27ac ChIP-Seq signals at all H3K27ac peaks. Color intensity of each dot corresponds to Lsd1 ChIP-Seq signal intensity. Lsd1 ChIP-Seq data are from *n* = 1 biological replicate from pooled islets of ad libitum–fed mice. Data were highly correlated with Lsd1 ChIP-Seq data from an independent biological replicate (see Supplemental Table 1). (**B**) Proportion of Lsd1 peaks associated with the indicated classes of H3K27ac peaks ± 1 kb. Numbers indicate Lsd1 peaks associated with each class of H3K27ac peaks. (**C**) Enrichment test of association frequencies between Lsd1 peaks and the indicated classes of H3K27ac peaks ± 1 kb. White bars indicate association frequencies expected by chance. ***P < 0.001 for enrichment; ^###^*P* < 0.001 for depletion by permutation test. (**D**) Enrichment of average ChIP-Seq signal for the indicated TFs at H3K27ac peaks gaining acetylation with feeding and associated with an Lsd1 peak ± 1 kb relative to static H3K27ac peaks associated with an Lsd1 peak ± 1 kb plotted against enrichment of average ChIP-Seq signal at the same sites relative to H3K27ac peaks gaining acetylation with feeding and not associated with an Lsd1 peak. (**E**) Histograms of ChIP-Seq signal for the indicated TFs at the indicated classes of H3K27ac peaks associated or not associated with an Lsd1 peak ± 1 kb. (**F**) Lsd1 ChIP-Seq signal for islets isolated from fed or fasted mice at the indicated classes of H3K27ac peaks. *n* = 2. Box plot whiskers span data points within the interquartile range × 1.5. *P < 0.05; ***P < 0.001, Wilcoxon’s rank-sum test corrected for multiple comparisons with the Benjamini-Hochberg procedure. (**G**) Lsd1 ChIP-Seq genome browser tracks for the indicated genes.

Based on the known role of Lsd1 in coupling nutrient state to gene expression by regulating histone methylation and acetylation (21), we investigated whether Lsd1 associates with nutrient-regulated chromatin in islets. ChIP-Seq for Lsd1 in islets revealed a positive correlation between Lsd1 and H3K27ac signal intensities (Spearman’s σ = 0.84, *P* < 2.2 × 10^-16^; Figure 2A, Supplemental Table 1, and Supplemental Table 3B) and predominant association of Lsd1 with active chromatin (Supplemental Figure 2, A–C), as was observed in other cell types (19, 27). Analysis of the extent of overlap between Lsd1-bound sites and feeding-regulated active chromatin revealed enrichment of Lsd1 binding and signal intensity at sites gaining H3K27ac with feeding (Figure 2, B and C, Supplemental Figure 2, D and E, and Supplemental Table 3C). Supporting the relevance of these binding events for gene regulation, Lsd1 peaks were overrepresented near genes upregulated by feeding (Supplemental Figure 2F). Thus, Lsd1 occupies active chromatin sites that gain acetylation with feeding and associates with feeding-induced genes. Analysis of TF motif and ChIP enrichment of Lsd1-bound sites exhibiting feeding-induced gains in H3K27ac revealed enrichment of several signal-dependent TFs such as Creb (28), Foxo1 (24, 29), Rfx3 (30), and Srf (31) as well as their cognate motifs at these sites (Figure 2, D and E, Supplemental Figure 2G, and Supplemental Table 4, B and C). Enrichment of nutrient-regulated TFs at these sites raises the possibility that these TFs recruit Lsd1 to chromatin in a nutrient-dependent manner. To determine whether Lsd1 is dynamically recruited with changing nutrient states, we performed ChIP-Seq for Lsd1 in islets from fasted and fed mice. Quantification of Lsd1 binding to active chromatin revealed that Lsd1 recruitment is highest in the fasted state and most prominent at sites that gain H3K27ac with feeding (Figure 2F and Supplemental Figure 2, B and H). Therefore, Lsd1 recruitment during fasting is accompanied by deacetylation of feeding-regulated active chromatin. This fasting-stimulated recruitment of Lsd1 was observed for regulatory elements near feeding-induced genes such as *Irs2*, *Mapkapk3*, and *Pak3* (Figure 2G), suggesting Lsd1 could be involved in regulation of these nutrient signaling genes by feed-fast cycles.

### Lsd1 inactivation in β cells causes insulin hypersecretion and hypoglycemia.

**Figure 3:**
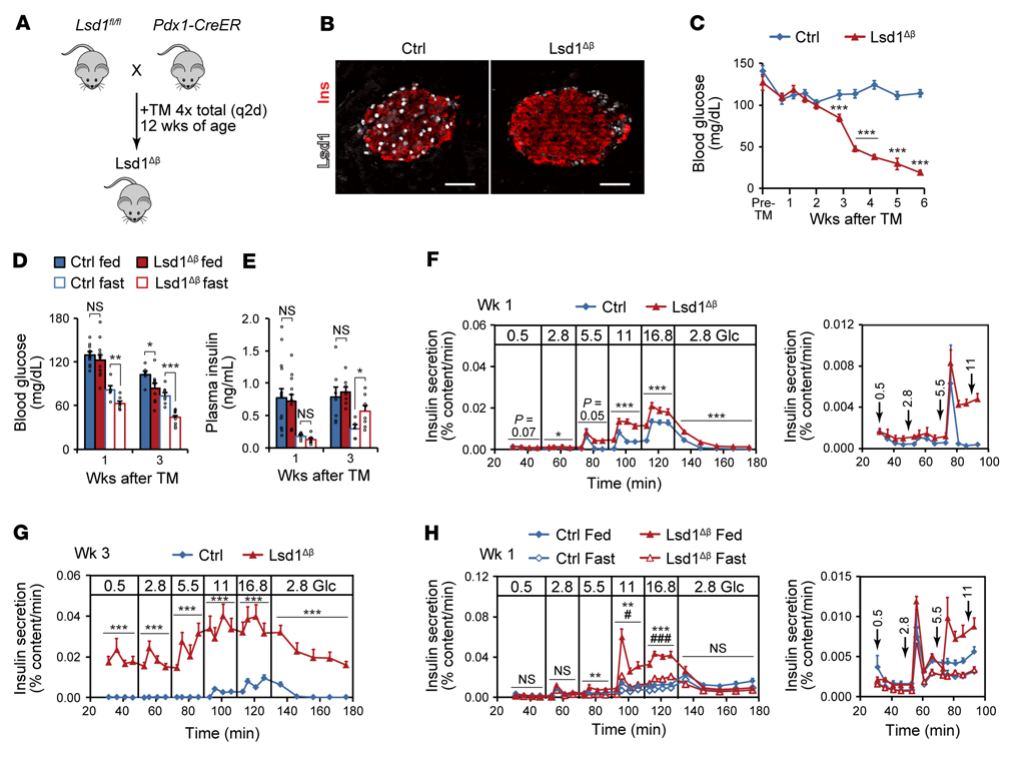
*Lsd1* inactivation in β cells causes insulin hypersecretion and hypoglycemia. (**A**) Schematic of alleles and treatments used to inactivate *Lsd1*. TM, tamoxifen; q2d, every other day. (**B**) Immunofluorescence staining for insulin (Ins) and Lsd1 on pancreas sections from control (control TM-treated *Lsd1^fl/+^*; *Pdx1-CreER*) and Lsd1^Δβ^ mice 2 days after TM treatment. Scale bars: 50 μm. (**C**) Time course of ad libitum–fed blood glucose levels in control (TM-treated *Lsd1^+/+^*; *Pdx1-CreER*) and Lsd1^Δβ^ mice. *n* = 9–10 mice. Pre-TM, within 3 days prior to initial TM injection. ***P < 0.001, unpaired 2-tailed *t* test. (**D** and **E**) Blood glucose (**D**) and serum insulin (**E**) levels in ad libitum–fed and 16 hour–fasted mice. *n* = 5–15 mice. *P < 0.05; **P < 0.01; ***P < 0.001, unpaired, 2-tailed *t* test. (**F** and **G**) Insulin secretion by control and Lsd1^Δβ^ islets during perifusion with the indicated glucose (Glc) concentrations (in mM) at 1 week (**F**) and 3 weeks (**G**) following TM treatment. *n* = 4 pools of 130 islets. Right (**F**), data shown at a reduced scale. *P < 0.05; ***P < 0.001, 2-way ANOVA for genotype for each time block. (**H**) Insulin secretion by control and Lsd1^Δβ^ islets during perifusion with the indicated glucose concentrations (in mM) from mice that were feeding entrained and then fed or fasted as in Supplemental Figure 1A. *n* = 3 pools of 130 islets. Right, data shown at a reduced scale for the indicated time points. **P < 0.01; ***P < 0.001, 2-way ANOVA for genotype between fed islets for each time block. ^#^*P* < 0.05; ^###^*P* < 0.001, 2-way ANOVA for genotype between fasted islets for each time block. Data are represented as mean ± SEM.

To investigate the function of Lsd1 in β cells, we deleted *Lsd1* in β cells of adult mice using *Pdx1-CreER* (Lsd1^Δβ^ hereafter; Figure 3, A and B, and Supplemental Figure 3A) and monitored glucose homeostasis. Ad libitum–fed male and female Lsd1^Δβ^ mice began to exhibit hypoglycemia 3 weeks after *Lsd1* inactivation (Figure 3C and Supplemental Figure 3B). The same phenotype was observed following *Lsd1* deletion with the *MIP-CreER* transgene (Supplemental Figure 3C) or catalytic inactivation of Lsd1 (32) in β cells (Lsd1^KI/KIβ^ hereafter), albeit with some delay (Supplemental Figure 3, D–F). Lsd1^Δβ^ and control mice did not differ regarding food consumption or body weight, and insulin sensitivity was modestly reduced (Supplemental Figure 3, G–I), excluding reduced caloric intake or increased insulin sensitivity as the cause of hypoglycemia. Analysis of β cell mass, islet endocrine cell-type composition, and pancreatic insulin content (Supplemental Figure 3, J–L) further ruled out β cell hyperplasia as underlying the hypoglycemia.

To investigate the progressive nature and possible nutrient dependency of the hypoglycemic phenotype, we analyzed blood glucose levels in Lsd1^Δβ^ mice in different nutritional states before and at the onset of overt hypoglycemia. One week after *Lsd1* deletion, ad libitum–fed mice maintained normal blood glucose levels throughout the day (Supplemental Figure 3M). A glucose tolerance test further indicated that glucose sensing during stimulation is normal 1 week following *Lsd1* inactivation (Supplemental Figure 3N). However, after overnight fasting, Lsd1^Δβ^ mice exhibited significantly lower blood glucose levels (Figure 3D). Fasting hypoglycemia was accompanied by inappropriately high plasma insulin levels in the fasted state (Figure 3E), which was also observed in Lsd1^KI/KIβ^ mice (Supplemental Figure 3O). It is unlikely that this phenotype is caused by a defective counterregulatory response because blood glucagon levels were not reduced in fasted Lsd1^Δβ^ mice (Supplemental Figure 3P). These findings suggest that the ability to suppress insulin secretion in the fasted state is eventually disrupted after *Lsd1* inactivation.

To determine whether Lsd1^Δβ^ mice exhibit insulin hypersecretion at substimulatory glucose concentrations, we studied Lsd1^Δβ^ islets in perifusion experiments. One week after *Lsd1* deletion, Lsd1^Δβ^ islets exhibited increased insulin secretion at intermediate and high glucose concentrations compared with control islets (Figure 3F), and by the 3-week time point, islets secreted insulin even at supraphysiologically low glucose levels (Figure 3G). Similar dysregulation of basal insulin secretion was observed in Lsd1^KI/KIβ^ mice (Supplemental Figure 3Q). Lsd1^Δβ^ islets hypersecrete insulin in response to glucose prior to an overt in vivo phenotype, suggesting transient in vivo compensation. Confirming this, in vitro hypersecretion occurred immediately after in vitro *Lsd1* deletion (Supplemental Figure 3, R and S). These results demonstrate that *Lsd1* inactivation results in cell-autonomous changes to insulin secretion first involving increased GSIS, followed by failure to suppress insulin secretion in response to hypoglycemia and blunting of additional glucose-induced increases.

The nutrient-dependent recruitment of Lsd1 to feeding-regulated chromatin in islets (Figure 2, F and G. and Supplemental Figure 2, B and H) suggests Lsd1 could have context-dependent functions related to nutrient state. We therefore tested the ability of Lsd1^Δβ^ islets to adapt GSIS to feeding and fasting. Control and Lsd1^Δβ^ mice were feeding entrained then refed or fasted as in Supplemental Figure 1A (Supplemental Figure 3T); then islets were isolated and insulin secretion was assessed. While the insulin-secretory response was similar between control and Lsd1^Δβ^ islets isolated from fasted mice, Lsd1^Δβ^ islets exhibited profound insulin hypersecretion in response to feeding (Figure 3H). This experiment demonstrates that Lsd1 has a context-specific role in dampening insulin secretion during physiological adaptation to feeding.

Insulin secretion is modulated by fatty acids and amino acids as well as hormones (3); therefore, we tested to determine whether islets from Lsd1^Δβ^ mice respond aberrantly to other secretagogues. While Lsd1^Δβ^ islets responded normally to the fatty acid palmitate and to costimulation with leucine and glutamine (Supplemental Figure 4A), they exhibited an exaggerated response to Exendin-4, an analogue of the feeding-induced hormone Glp-1 (Supplemental Figure 4B). The increased sensitivity to Glp-1 or other cAMP-generating pathways (2) could contribute to hypoglycemia in the fed state following *Lsd1* inactivation (Figure 3D).

### Accelerated glucose metabolism promotes insulin hypersecretion in Lsd1^Δβ^ islets.

**Figure 4:**
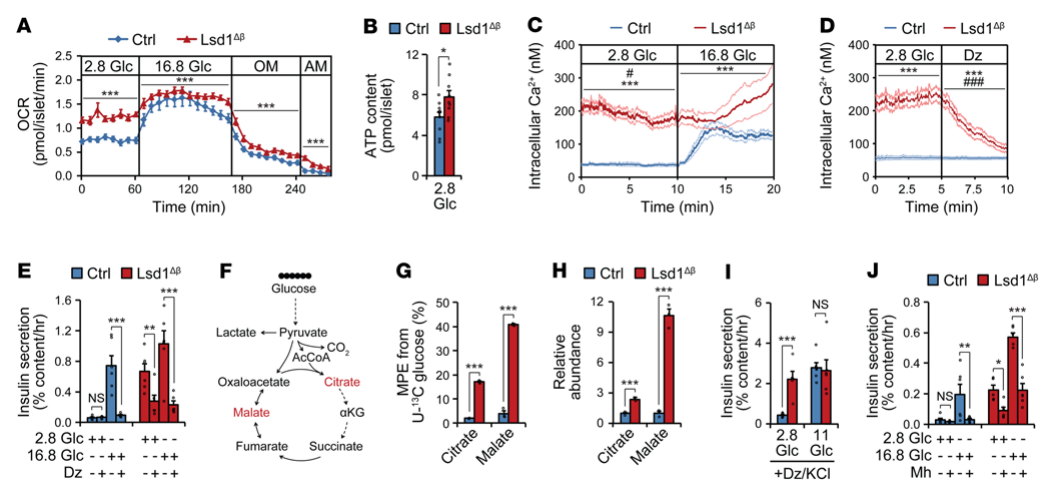
Accelerated glucose metabolism promotes insulin hypersecretion in Lsd1^Δβ^ islets. (**A**) Oxygen consumption rate (OCR) of islets treated sequentially with the indicated glucose concentrations (in mM), oligomycin (OM), and antimycin A (AM). *n* = 8–10 pools of 60 islets. ***P < 0.001, 2-way ANOVA for genotype for each time block. (**B**) ATP content of islets. *n* = 9–10 pools of 20 islets. *P < 0.05, unpaired 2-tailed *t* test. (**C** and **D**) Intracellular Ca^2+^ concentration of β cells treated with glucose (in mM) (**C**) or the K_ATP_ channel opener diazoxide (Dz) (**D**). *n* = 32–46 β cells, representative of 3 independent experiments. ***P < 0.001, 2-way ANOVA for genotype for each time block. ^#^*P* < 0.05; ^###^*P* < 0.001, 2-way ANOVA for the interaction between genotype and time for each time block. (**E**) Insulin-secretion by islets with and without Dz. *n* = 5–6 pools of 10 islets. **P < 0.01; ***P < 0.001, pairwise *t* test corrected for multiple comparisons with the Benjamini-Hochberg procedure following 2-way ANOVA for genotype and stimulation condition. (**F**) Schematic of tracing experiment. (**G** and **H**) Molar percentage enrichment (MPE) of ^13^C (**G**) and relative abundances (**H**) of indicated metabolites after tracing with 2.8 mM U-^13^C glucose. *n* = 3 pools of 220 islets. ***P < 0.001, unpaired 2-tailed *t* test. (**I** and **J**) Insulin secretion by islets under depolarizing conditions (30 mM KCl and 100 μM Dz) (**I**) or with and without the glycolysis inhibitor mannoheptulose (Mh) (**J**). *n* = 5–6 pools of 10 islets. *P < 0.05; **P < 0.01; ***P < 0.001, pairwise *t* test corrected for multiple comparisons with the Benjamini-Hochberg procedure following 2-way ANOVA for genotype and stimulation condition. Islets were isolated from ad libitum–fed animals 3 weeks following tamoxifen treatment. Data are represented as mean ± SEM.

β Cell glucose sensing relies on glucose metabolism, such that the rate of glycolysis is determined by blood glucose concentration. An increase in the β cell ATP/ADP ratio in response to glucose triggers insulin secretion by inducing closure of an ATP-sensitive potassium (K_ATP_) channel, membrane depolarization, and subsequent calcium (Ca^2+^) influx (4). Having found that insulin secretion becomes progressively uncoupled from glucose levels in *Lsd1*-deficient β cells, we sought to determine which step or steps of stimulus-secretion coupling are deregulated. As a readout for metabolic activity, we measured respiration under basal and stimulatory glucose conditions (2.8 mM and 16.8 mM glucose, respectively) and found that Lsd1^Δβ^ islets consumed more oxygen in low glucose than control islets (Figure 4A). Reduced respiration following inhibition of ATP synthase by oligomycin indicates that oxygen consumption in Lsd1^Δβ^ islets is coupled to ATP synthesis (Figure 4A). Accordingly, ATP content in low glucose was elevated in Lsd1^Δβ^ islets (Figure 4B). To study stimulus-secretion coupling downstream of ATP production in Lsd1^Δβ^ islets, we measured Ca^2+^ influx in basal and stimulatory glucose concentrations. Consistent with their higher ATP content, *Lsd1*-deficient β cells exhibited elevated Ca^2+^ levels in basal glucose levels akin to those of control β cells under stimulatory glucose concentrations (Figure 4C), suggesting constitutive activation of voltage-gated Ca^2+^ channels. To determine whether Ca^2+^ influx in Lsd1^Δβ^ β cells is triggered by tonic inhibition of K_ATP_ channels in basal glucose concentrations, we treated islets with the K_ATP_ channel opener diazoxide. Showing the K_ATP_ channel dependency of Ca^2+^ influx in *Lsd1*-deficient β cells, diazoxide inhibited Ca^2+^ influx (Figure 4D) and reduced basal insulin secretion (Figure 4E). Together, these results suggest that altered metabolism in Lsd1^Δβ^ islets promotes basal insulin hypersecretion via closure of K_ATP_ channels and subsequent activation of voltage-gated Ca^2+^ channels.

To directly test whether glucose metabolism fuels ATP production and constitutive insulin secretion in Lsd1^Δβ^ islets, we determined the metabolic fate of glucose in islets using isotopic tracing of uniformly ^13^C-labeled glucose (hereafter U-^13^C glucose; Figure 4F). Control and Lsd1^Δβ^ islets were incubated in 2.8 mM of U-^13^C glucose, reflective of a nonstimulatory glucose concentration, and analyzed by targeted metabolomics to determine abundance and isotopic labeling of glucose-derived metabolites. Lsd1^Δβ^ islets exhibited increased isotopic labeling and accumulation of citrate and malate (Figure 4, G and H), which have been shown to increase during glucose stimulation (33, 34). Lsd1^Δβ^ islets also exhibited increased ^13^C labeling of glycine, aspartate, and glutamate (Supplemental Figure 4C), reflecting accelerated glucose metabolism via glycolysis and the TCA cycle. Lactate production was not altered (Supplemental Figure 4D), suggesting that Lsd1^Δβ^ islets metabolize much of this glucose in mitochondria. These results indicate that *Lsd1* deficiency establishes a basal metabolic state that resembles the glucose-stimulated state of normal islets.

Glucose metabolism stimulates insulin secretion via both “triggering” and “amplifying” pathways (4). The triggering pathway is stimulated when ATP produced from glucose inhibits K_ATP_ channels, resulting in depolarization and opening of voltage-gated Ca^2+^ channels. The amplifying pathway potentiates the effect of Ca^2+^ upon insulin exocytosis, which occurs in part via accumulation of intermediary metabolites, including citrate and malate (3, 34). We assessed activity of the amplifying pathway by constitutively activating the triggering pathway with KCl and preventing metabolic effects on the K_ATP_ channel with simultaneous diazoxide treatment. This revealed constitutive activation of the amplifying pathway in Lsd1^Δβ^ islets (Figure 4I). Supporting the important role of accelerated glucose metabolism in basal insulin hypersecretion by *Lsd1*-deficient β cells, inhibition of glycolysis with mannoheptulose partially rescued basal insulin hypersecretion by Lsd1^Δβ^ islets (Figure 4J). Together, these findings suggest that aberrant glucose metabolism in the unstimulated state activates both triggering and amplifying pathways of insulin secretion in Lsd1^Δβ^ islets.

### Lsd1 inactivation in β cells deregulates genes involved in nutrient signaling and cell metabolism.

**Figure 5:**
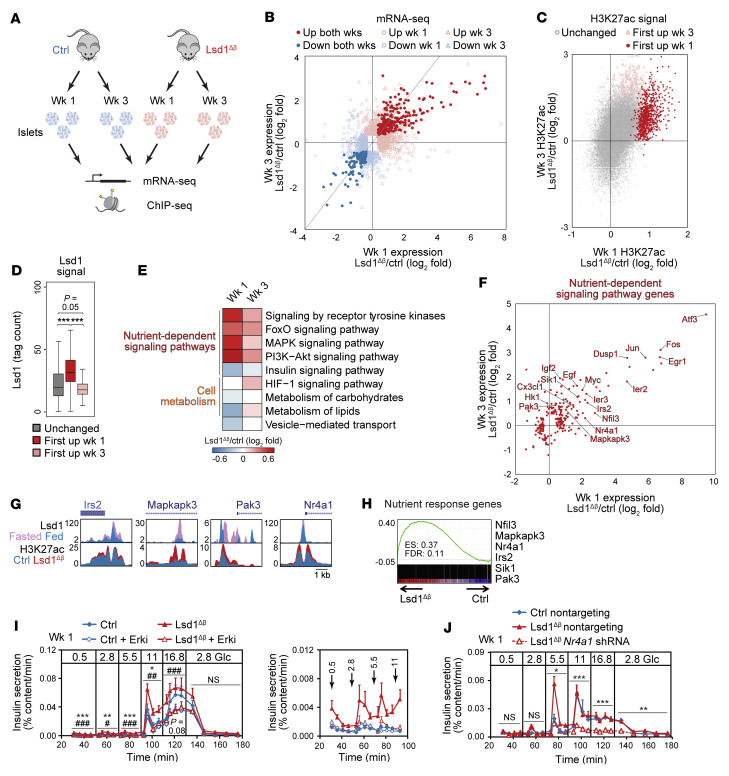
*Lsd1* inactivation in β cells deregulates genes involved in nutrient-dependent signaling and cell metabolism. (**A**) Schematic of experiments. wk/s, week/s. (**B**) Relative mRNA levels for differentially expressed genes (*P* < 0.01 by Cuffdiff). Gray line indicates slope of 1. *n* = 3–5. (**C**) Relative H3K27ac ChIP-Seq signal at H3K27ac peaks. *n* = 2. (**D**) Lsd1 ChIP-Seq signal at classes of H3K27ac peaks. Lsd1 ChIP-Seq data are from *n* = 1 replicate, highly correlated with an independent biological replicate, from islets of ad libitum–fed mice. Box plot whiskers span data points within the interquartile range × 1.5. ***P < 0.001, Wilcoxon’s rank-sum test corrected for multiple comparisons with Benjamini-Hochberg procedure. (**E**) Median log_2_ fold expression changes for functional categories. (**F**) log_2_ fold changes for differentially expressed genes (*P* < 0.01 by Cuffdiff) annotated to nutrient-dependent signaling pathways. *n* = 3–5. (**G**) ChIP-Seq genome browser tracks Lsd1^Δβ^ islet data shown at 1 week following TM treatment. (**H**) GSEA of nutrient-response genes (from Figure 1I) against mRNA-Seq data from Lsd1^Δβ^ islets 1 week following TM treatment. (**I** and **J**) Insulin secretion by islets during perifusion with the indicated glucose concentrations (in mM) following 24-hour treatment with SCH772984 (Erki) or vehicle (**I**) or following transduction with shRNAs (**J**). Right (**I**), data shown at a reduced scale. *n* = 6 pools of 130 islets (**I**) or *n* = 3 pools of 220 reaggregated islets (**J**). Data are represented as mean ± SEM. *P < 0.05; **P < 0.01; ***P < 0.001 by 2-way ANOVA for genotype in vehicle-treated islets for each time block. ^#^*P* < 0.05; ^##^*P* < 0.01; ^###^*P* < 0.001, Erki-treated relative to vehicle-treated Lsd1^Δβ^ islets by 2-way ANOVA for treatment group for each time block (**I**). *P < 0.05; **P < 0.01; ***P < 0.001, by 2-way ANOVA for shRNA for each time block (**J**).

Having observed altered insulin secretion in Lsd1^Δβ^ mice, we sought to determine the underlying molecular mechanisms. To this end, we characterized transcriptomes and epigenomes of Lsd1^Δβ^ islets before and at the onset of hypoglycemia at 1 and 3 weeks after *Lsd1* deletion, respectively (Figure 5A). mRNA-Seq analysis revealed that many transcriptional changes observed 3 weeks after *Lsd1* inactivation were already present at week 1 (Spearman’s σ = 0.49, *P* < 2.2 × 10^-16^ for regulated genes; Figure 5B, Supplemental Table 1, and Supplemental Table 2C). Thus, loss of *Lsd1* in β cells has effects on gene transcription prior to manifestation of overt hypoglycemia. These gene expression changes were largely recapitulated by catalytic inactivation of Lsd1 in β cells (Supplemental Figure 5A and Supplemental Table 2C). Concordant with the transcriptomic changes, Lsd1^Δβ^ islets also exhibited early alterations in the islet chromatin state (Figure 5C, Supplemental Figure 5B, Supplemental Table 1, and Supplemental Table 3D). Catalytic inactivation of Lsd1 resulted in changes to H3K27ac similar to those with *Lsd1* deletion in β cells (Supplemental Figure 5, B and C). Early H3K27ac changes following *Lsd1* deletion comprised almost exclusively a gain in acetylation (717 differential peaks gaining versus 1 peak losing acetylation at week 1). Among all classes of H3K27ac sites, early hyperacetylated sites exhibited the strongest Lsd1-binding signal (Figure 5D), suggesting direct effects of Lsd1. These sites also exhibited H3K4me1 accumulation (Supplemental Figure 5, D–G, and Supplemental Table 3E), consistent with Lsd1’s activity as a H3K4 mono- and didemethylase (35).

To identify gene expression changes that drive insulin hypersecretion following *Lsd1* inactivation, we determined functional category enrichment among differentially expressed genes in Lsd1^Δβ^ islets. Enrichments were independently assessed for genes regulated at 1 or 3 weeks after *Lsd1* inactivation to account for transient or delayed regulation (Supplemental Table 2, C and E–G). This analysis revealed deregulation of processes involved in the control of insulin secretion, such as nutrient-dependent signaling, cell metabolism, and vesicle transport (Figure 5, E and F, and Supplemental Figure 5, H and I). Nutrient-dependent signaling pathway genes were also upregulated by catalytic inactivation of Lsd1 in β cells, indicating that regulation of these genes occurs through Lsd1’s demethylase activity (Supplemental Figure 5J). Among the deregulated genes were ligands that stimulate receptors of nutrient-responsive signaling pathways (*Cx3cl1*, *Egf*, and *Igf2*) as well as downstream signaling intermediates for these pathways (*Irs2*, *Mapkapk3*, and *Pak3*) and nutrient-regulated TFs (*Atf3*, *Fos,* and *Nr4a1*), which all have positive effects on insulin secretion (11, 36–40). Active chromatin proximal to many nutrient-response genes, such as *Irs2*, *Mapkapk3*, *Nr4a1*, and *Pak3* (Figure 5G and Supplemental Figure 5K), was Lsd1 bound and exhibited H3K27 hyperacetylation and increased H3K4 monomethylation in Lsd1^Δβ^ islets, indicating direct effects of Lsd1 on the chromatin state at these gene loci. Dysregulation of carbohydrate metabolism genes (e.g., *Pfkfb2*) and HIF-1 pathway genes (*Egfr*, *Hk1*, *Igf1*, and *Pfkfb3*) suggests *Lsd1* deletion could lead to metabolic remodeling (Supplemental Table 2, F and G). Of note, the low-K_m_ glucose-phosphorylating enzyme hexokinase 1 (*Hk1*), which is normally repressed in β cells (41), was only upregulated at 3 weeks, but not at 1 week after *Lsd1* inactivation (Figure 5F). Ectopic Hk1 expression in β cells accelerates glycolysis in low glucose and causes basal insulin hypersecretion and hypoglycemia (42) similar to that in Lsd1^Δβ^ mice 3 weeks after *Lsd1* inactivation (Figure 3, D and G). Examination of other genes selectively repressed in islets or β cells (“disallowed” genes) (43–45) revealed upregulation of a subset of these genes following β cell *Lsd1* inactivation (Supplemental Figure 6, A and B). Notably, disallowed genes involved in lactate production and export (Ldha and Slc16a1) were not upregulated in Lsd1^Δβ^ islets, indicating distinct mechanisms of repression for subsets of disallowed genes (Supplemental Figure 6B). Given the involvement of several disallowed genes in establishing the unique metabolic program of β cells, it is possible that these changes contribute to altered metabolism in Lsd1^Δβ^ islets through as-yet-undefined gene functions. Taken together, these observations support a model whereby *Lsd1* inactivation deregulates nutrient-dependent signaling pathways and leads to metabolic reprogramming of β cells, thereby leading to aberrantly high insulin secretion in response to feeding or in basal glucose.

### Lsd1 inactivation uncouples histone acetylation and nutrient-response gene expression from feeding state.

The similarities between effects of *Lsd1* deletion (Figure 5C), short-term feeding (Figure 1D), and chronic overfeeding (Figure 1J) on islet H3K27ac raised the possibility that Lsd1 dampens acetylation at nutrient-regulated H3K27ac sites. Indeed, sites that gained H3K27ac with feeding exhibited excess H3K27ac deposition in both Lsd1^Δβ^ and Lsd1^KI/KIβ^ islets and did so to a greater extent than sites not regulated by feeding (Supplemental Figure 7, A and B). Uncoupling of histone acetylation from the feeding state in Lsd1^Δβ^ islets indicates that Lsd1 is required for the β cell to interpret nutrient signals at the level of the epigenome. Lsd1^Δβ^ (Supplemental Figure 5K and Supplemental Figure 7C) and *db/db* islets (Supplemental Figure 1M) both exhibited increased H3K4 monomethylation at feeding-induced H3K27ac sites, whereas a gain in H3K4me1 was not observed after short-term feeding (Supplemental Figure 1H and Supplemental Figure 2B). Thus, *Lsd1* deletion or prolonged overfeeding have more pronounced effects on the epigenome relative to short-term feeding.

The overlap in cellular processes affected by *Lsd1* deletion (Figure 5E) and induced by feeding (Figure 1C) led us to examine whether *Lsd1* inactivation deregulates genes similar to those regulated during adaptation to changing nutrient states. GSEA revealed enrichment of nutrient-response genes among genes overexpressed in Lsd1^Δβ^ islets (Figure 5H and Supplemental Figure 7, D and E). Similar genes were acutely induced in β cells (10) and islets by costimulation with glucose and the Glp-1 effector cAMP (Supplemental Figure 7, F and G). Target genes regulated by Lsd1 converged with those of several nutrient-regulated TFs that cobind with Lsd1 in the genome, including Foxo1 (46), Creb (28), and Srf (47) (Figure 2, D and E, and Supplemental Figure 7H). To directly determine whether Lsd1 is required for feeding-induced gene regulation in β cells, we analyzed islet transcriptomes of control and Lsd1^Δβ^ mice subjected to fasting and refeeding, as in Figure 3H. *Lsd1*-deficient β cells exhibited a reduced amplitude of feeding-induced transcriptional changes (Supplemental Figure 7, I and J), indicating a requirement for Lsd1 in β cell transcriptional adaptations to feeding. Together, these findings show that Lsd1 mediates coupling of environmental nutrient signals to transcription of genes associated with β cell functional adaptation.

The prevalence of nutrient-response genes with known roles in insulin secretion (e.g., *Atf3*, *Irs2*, and *Nr4a1*) (31, 38, 39) among Lsd1-regulated transcripts (Figure 5, F and H) suggested that Lsd1 could adapt insulin secretion by modulating nutrient-dependent signaling. Notably, both feeding and *Lsd1* deletion stimulated expression of MAPK pathway–associated genes (Figure 1C and Figure 5E). To determine the importance of MAPK signaling for insulin hypersecretion in Lsd1^Δβ^ islets, we treated control and Lsd1^Δβ^ islets with an ERK inhibitor for 24 hours. Indeed, ERK inhibitor treatment rendered the insulin-secretory response of Lsd1^Δβ^ islets indistinguishable from that of control islets (Figure 5I). Together, these results demonstrate that inhibition of MAPK signaling reverses insulin hypersecretion induced by *Lsd1* inactivation, indicating functional convergence of Lsd1-regulated programs and the MAPK-signaling pathway in β cells.

We then sought to determine direct Lsd1 target genes that mediate the effect of *Lsd1* inactivation on GSIS. We focused on *Nr4a1* (Figure 5G and Supplemental Figure 5K), which is upregulated in response to feeding or *Lsd1* inactivation (Figure 5F) and promotes insulin secretion (39). Indeed, *Nr4a1* knockdown by shRNA reduced GSIS by Lsd1^Δβ^ islets (Figure 5J and Supplemental Figure 7K), suggesting that feeding promotes adaptive insulin secretion in part through *Nr4a1* upregulation, which is normally restrained by Lsd1.

### LSD1 regulates insulin secretion in human islets.

**Figure 6:**
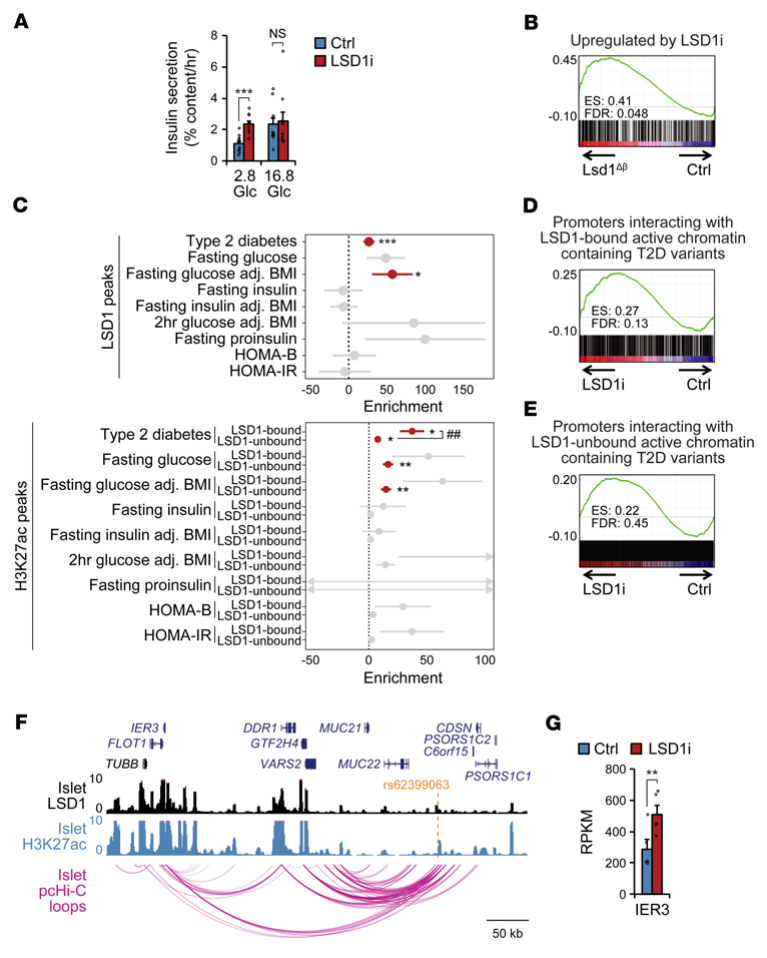
LSD1 regulates insulin secretion in human islets, and its binding sites are enriched for T2D-associated variants. (**A**) Static insulin-secretion assay in human islets stimulated with the indicated glucose concentrations (in mM) following 24-hour treatment with the LSD1i TCP or vehicle control. *n* = 8 donors. Data are represented as mean ± SEM. ***P < 0.001, paired, 2-tailed *t* test. (**B**) GSEA of mouse orthologs of genes upregulated by LSD1i treatment in human islets (*P* < 0.01 by Cuffdiff; *n* = 5 donors) against mRNA-Seq data from Lsd1^Δβ^ and control mouse islets 1 week following TM treatment. (**C**) GWAS enrichment (%*h^2^*/%SNPs) of metabolic traits at LSD1 ChIP-Seq peaks (from *n* = 2 donors, top) and H3K27ac ChIP-Seq peaks associated with an LSD1 peak ± 1 kb or not associated with an LSD1 peak (bottom) in human islets using LD-score regression. Data shown represent enrichment estimate and SEM, and significant estimates are highlighted in red. *P < 0.05; **P < 0.01; ***P < 0.001 (Bonferroni-corrected) for enrichment by LD-score regression. ^##^*P* < 0.01 for differences in enrichment between LSD1-bound and -unbound active chromatin by 2-tailed Welch’s *t* test. (**D** and **E**) GSEA of genes whose promoters interact with an LSD1-bound (**D**) or LSD1-unbound (**E**) site containing a T2D-associated variant from pcHi-C data against mRNA-Seq data from LSD1i-treated and control human islets. (**F**) LSD1 and H3K27ac ChIP-Seq and pcHi-C (17) genome browser tracks from human islets. (**G**) Bar graph of *IER3* mRNA levels in human islets treated with LSD1i as in **A**. Data are represented as mean ± SEM. **P < 0.01 by Cuffdiff.

To determine whether LSD1 function is conserved in human β cells, we treated human islets with the LSD1 inhibitor tranylcypromine (LSD1i) or conducted knockdown in human β cells and performed GSIS assays. Similarly to Lsd1^Δβ^ islets (Supplemental Figure 4, A and B), LSD1i-treated human islets or β cells transduced with an *LSD1* shRNA exhibited increased basal insulin secretion (Figure 6A and Supplemental Figure 8, A–D). Transcriptome analysis of LSD1i-treated human islets revealed that genes induced by LSD1i treatment were also upregulated in Lsd1^Δβ^ mouse islets (Figure 6B), including nutrient-response genes (Supplemental Table 1 and Supplemental Table 2D).

### LSD1-binding sites are enriched for type 2 diabetes–associated variants.

To further determine whether LSD1 associates with active chromatin in human islets, we conducted ChIP-Seq for LSD1 (Supplemental Table 1 and Supplemental Table 3F). As in mouse islets (Figure 2, A and B, and Supplemental Figure 2, A and B), LSD1 predominantly occupied active promoters and enhancers in human islets (Supplemental Figure 8E and Supplemental Table 3F), with LSD1 and H3K27ac signal intensities correlating across the genome (Spearman’s σ = 0.79, *P* < 2.2 × 10^-16^; Supplemental Figure 8, F–H, and Supplemental Table 3G).

Given the here-demonstrated role for LSD1 in the regulation of insulin secretion, we postulated that genetic variants associated with traits relevant to insulin secretion could exert their function through LSD1-occupied sites. To test this, we calculated enrichment of genetic variants at LSD1-bound sites for association with type 2 diabetes (T2D) (48), diabetes-related quantitative phenotypes (49–52), and other complex traits and diseases for calibration (see Methods). We observed significant enrichment of T2D and BMI-adjusted fasting glucose (FG adj. BMI) association (Figure 6C). As expected, traits associated with insulin resistance (e.g., fasting insulin) as well as most nonmetabolic traits showed no evidence for enrichment (Supplemental Figure 8I). As islet enhancers are enriched for genetic variants associated with T2D and FG adj. BMI (53), we asked whether LSD1-bound islet active chromatin exhibits a stronger enrichment for variants associated with T2D and metabolic traits than islet active chromatin not bound by LSD1. Indeed, T2D-associated variants are enriched at LSD1-bound sites to a greater extent than at LSD1-unbound sites (*P* = 0.0056 by 2-tailed Welch’s *t* test; Figure 6C and Supplemental Figure 8I), indicating LSD1-bound active chromatin is specifically enriched for T2D-associated variants beyond what is expected for islet active chromatin in general.

To define the LSD1-regulated gene network associated with T2D risk variants, we linked LSD1-bound sites containing T2D variants to their putative target genes in islets using chromatin loops derived from promoter capture Hi-C (pcHi-C) data (17) (Supplemental Table 5, A and B). Genes looped to T2D-associated, LSD1-bound sites were significantly enriched among genes upregulated in LSD1i-treated islets (Figure 6, D and E). For example, T2D-associated variants in LSD1-occupied enhancers exhibited chromatin looping to the *IER3* and *JUN* promoters (Figure 6F and Supplemental Figure 8J). *IER3* and *JUN* mRNAs were upregulated by *LSD1* inactivation in both human and mouse islets (Figure 5F, Figure 6G, and Supplemental Figure 8K). Overall, these observations suggest T2D risk variants can influence the activity of the LSD1-regulated gene network in islets. In sum, our findings identify a key role for LSD1 in preserving glucose homeostasis of mice and humans by ensuring the insulin-secretory response is coupled to nutrient state.

## Discussion

Through integrated studies of β cell physiology, epigenomics, and transcriptomics, we establish nutrient-dependent regulation of the islet epigenome as a mechanism for β cell functional adaptation to changes in organismal insulin demand. We find that feeding induces histone hyperacetylation and transcription at gene loci involved in the regulation of β cell nutrient signaling and metabolism, indicating that nutrients sensitize the response of β cells to insulin secretory cues in part through effects on the epigenome. Our analysis identifies Lsd1 as a component of the transcriptional complexes residing at sites whose acetylation levels are coupled to nutrient state. By dampening histone acetylation and methylation at these sites, Lsd1 prevents aberrantly high expression of nutrient-induced genes, thereby counteracting the sensitization of the β cell insulin-secretory response by nutrient cues. The experimental uncoupling between nutrient state and regulation of the epigenome via *Lsd1* deletion results in a profound glucose homeostasis phenotype. Together, our findings identify the epigenome as a key regulatory layer of β cell function.

Metabolic tissues sense and respond to changes in nutrient state to preserve metabolic homeostasis despite fluctuations in energy availability. Immediate responses to a new nutrient state involve well-characterized endocrine and neuronal pathways that rapidly stimulate changes to nutrient utilization (1). Following an initial response, sustained changes in nutrient state evoke perduring tissue-intrinsic adaptations that adjust the function of metabolic tissues commensurate with the prevailing nutrient environment (16). Our data suggest that nutrient regulation of the epigenome adapts insulin-secretory responses by pancreatic β cells.

β Cell functional adaptations are exemplified by attenuation of the insulin-secretory response following a prolonged fast (5, 6) and sensitization of GSIS during chronic overnutrition (7, 8). Here, we demonstrate that β cell functional adaptation is already apparent 4 hours after feeding, indicating this response occurs over a much shorter time scale than was previously appreciated. This short-term effect is accompanied by changes to the epigenome and the transcriptome that we show are required for proportionally adjusting insulin secretion. We further demonstrate that the epigenome is affected by perturbations of metabolic homeostasis. In the *db/db* model of chronic overnutrition, we observed changes in H3K27 acetylation and gene expression that were qualitatively similar (but not identical) to those in the short-term feeding model at a time point when *db/db* islets exhibit adaptive insulin hypersecretion. These findings suggest that this epigenetic mechanism of nutrient-induced β cell functional adaptation is relevant during both short-term and chronic nutrient stimulation.

We show that Lsd1 function is coupled to nutrient state in part through its recruitment to and eviction from chromatin. The exact mechanism whereby Lsd1’s demethylase activity regulates gene expression in β cells is currently unclear. It is well documented that there is functional cooperativity between Lsd1 as a demethylating enzyme and deacetylating enzymes (54, 55), providing a potential link between *Lsd1* inactivation and altered histone acetylation including, but not limited, to H3K27ac. Moreover, it remains possible that Lsd1 regulates H3K27ac through demethylation of TFs or other nonhistone proteins. Together, our observations build a model showing that feeding-induced histone acetylation in β cells is reversed by recruitment of Lsd1 to chromatin in the fasted state (Supplemental Figure 8L). We speculate that nutrient regulation of the epigenome via Lsd1 is a mechanism for β cells to modulate a broad gene regulatory program that adjusts insulin secretion beyond the capabilities of any individual TF. Compellingly, a similar mechanism has been proposed for metabolic regulation of histone acetylation in yeast, wherein several nutrient-sensing TFs participate in remodeling of the epigenome via nutrient-regulated interactions with histone acetyltransferase–containing complexes (56).

Adaptation of metabolic tissues to changing nutrient states requires accurate interpretation of environmental signals. We show that in β cells, the chromatin modifier Lsd1 is required for interpreting environmental nutrient signals at the level of the epigenome. Gene-environment interactions play an important role in T2D pathogenesis (57). The observed enrichment of T2D-associated variants at LSD1-bound sites in human islets suggests that interpretation of nutrient signals is influenced by genetic variation, thereby having an impact on T2D risk. Our findings support a model whereby influences from environmental nutrient signals and genetic variation converge at the level of the β cell epigenome to affect adaptation of the insulin-secretory response. Deeper investigation of environmental regulation of the epigenome in metabolic tissues should have relevance for understanding the pathogenesis of T2D and should help pave the way for therapeutic intervention.

## Methods

### Animal studies.

All mice used were of mixed strain backgrounds with approximately equal contributions from C57BL/6N and CD1, with the exceptions of *db*/*db* mice (C57BLKS/J) and mice used for Lsd1 ChIP-Seq (C57BL/6N). Unless otherwise indicated, animals were maintained under standard housing conditions, and male mice were used for all experiments. The following mouse strains were used in this study: *Lsd1^fl^* (58), *Lsd1^KI^* (32), *Pdx1-CreER* (59), *MIP-CreER* (60), *mIns1-H2b-mCherry* (61), and *db*/*db* (Jackson Laboratory, strain 000642).

### Metabolic studies.

Feeding entrainment was performed as described previously (22), with the exception that the feeding period encompassed the entire 12-hour dark phase. Unless otherwise indicated, blood glucose (Bayer Contour glucometer), body weight, and serum hormones (ALPCO, Insulin ELISA, EMD Millipore, glucagon radioimmunoassay) were measured between ZT3 and ZT4. Food intake was measured by weighing food hoppers. Glucose and insulin tolerance tests were performed in mice fasted for 6 hours as described (62). Immunohistochemistry, islet endocrine cell composition, and pancreatic insulin content or β cell mass measurements were performed as described (63).

### Cell line and islet culture.

Mouse islets were isolated and cultured as described (33). For cAMP stimulation, islets were treated with 0.5 mM cpt-cAMP (Enzo) in media containing 11 mM glucose. For in vitro *Lsd1* deletion, islets were treated with 2 μM 4-hydroxy-tamoxifen or vehicle control (ethanol) diluted in islet media; then islets were harvested 72 or 96 hours later for Western blotting or perifusion, respectively.

Human islets were acquired through the Integrated Islet Distribution Program (Supplemental Table 6). Upon receipt, islets were stained with dithizone, then allowed to recover 18 to 48 hours in complete media (CMRL 1066 with 13 mM glucose, 10% FBS, 2 mM l-glutamine, 100 U/mL penicillin/streptomycin, 1 mM sodium pyruvate, 10 mM HEPES, and 0.25 mg/mL amphoterecin B). For LSD1 inhibition studies, 2 mM tranylcypromine (MilliporeSigma) or vehicle (H_2_O) was included in the culture media for 24 hours prior to analysis. Min6 insulinoma cells were cultured as described (11).

### Insulin-secretion measurements.

Static insulin-secretion assays were performed as described (33) following recovery from isolation or shipment or immediately following islet isolations for comparison of islets in the fed and fasted states and for comparison of *db/db* and control (*db/+*) islets.

For perifusion, islets were first starved for 30 minutes in Krebs-Ringer-bicarbonate-HEPES (KRBH) buffer (33) containing 2.8 mM glucose at 37°C with 5% CO_2_ and were then loaded into perifusion chambers. Once loaded, islets were equilibrated with KRBH containing 2.8 mM glucose for 30 minutes, at which point perifusate was collected for analysis. At the end of each experiment, islets were lysed by sonication for insulin-content determination.

### Islet respirometry.

Respirometry was performed using the Seahorse Bioscience XF24 Extracellular Flux Analyzer with 60 islets per well as described (33).

### ATP measurements.

Islet ATP measurements were performed using the Enliten ATP Assay Kit (Promega) as described (63).

### Calcium imaging.

Calcium imaging was performed with the indicated genotypes of mice also carrying the *mIns1-H2b-mCherry* transgene (61). Islets were dispersed with 0.05% trypsin/EDTA (Invitrogen), cultured overnight on glass-bottom dishes coated with poly-l-lysine, then loaded with Fluo-4. Fluorescence was monitored and analyzed as described (64).

### Targeted metabolomics.

Islets were prepared for targeted metabolomics essentially as described (33) with the following modifications. Islets were briefly washed in KRBH containing 2.8 mM unlabeled glucose, then transferred to KRBH containing 2.8 mM U-^13^C and incubated at 37°C and 5% CO_2_ for 2 hours to allow for ^13^C incorporation into downstream metabolites, which is not expected to result in steady-state labeling (33).

### Human β cell purification, lentiviral shRNA transduction, and insulin-secretion assays.

Immediately following shipment, human islets were dissociated with Versene (Thermo); then live β cells were FACS-sorted based on negativity for propidium iodide and HIC3-2D12 and positivity for HIC1-2B4, as described (65). Collected β cells were seeded into V-bottom 96-well plates and exposed overnight to pLKO.1-encoded lentiviruses producing either nontargeting shRNA or a pool of lentiviruses producing *LSD1* shRNA (Supplemental Table 7). Four days later, islet aggregates were starved for 1 hour in KRBH supplemented with 2.8 mM glucose, then were incubated with KRBH containing either 2.8 mM glucose or 16.8 mM glucose for 1 hour, after which insulin content of media and lysates was determined as described above. Knockdown was verified in whole dissociated islets infected as above and reaggregated in AggreWell plates (STEMCELL Technologies), using GFP-expressing pLKO.1-encoded lentivirus for visualization of transduction efficiency in control islets 72 hours following transduction.

### Mouse islet lentiviral shRNA transduction.

Islets were dissociated with StemPro Accutase (Thermo) and seeded into AggreWell plates in the presence of pLKO.5-encoded lentiviruses producing either nontargeting control shRNA or a pool of lentiviruses producing *Nr4a1* shRNA (Supplemental Table 7). Aggregated islet cells were harvested 3 or 4 days later for RNA or perifusion assays, respectively.

### RNA extraction, mRNA-Seq, and RT-qPCR.

RNA was isolated and quantitative reverse-transcription PCR (RT-qPCR) was performed as described (63) using primers listed in Supplemental Table 8. mRNA-Seq libraries were prepared from 35 ng of total RNA using the TruSeq Stranded mRNA Library Prep Kit (Illumina) with the exception of *db/db* mouse islets and their respective controls, from which libraries were generated as described (66). Libraries were single-end sequenced at a length of 50 bp or 75 bp per read using HiSeq 4000 or NovaSeq 6000 (Illumina), respectively.

### ChIP-Seq.

Islets were processed for ChIP immediately following isolation or shipping. For Lsd1 ChIP of human islets or ad libitum–fed mouse islets, islets were processed and ChIP was performed as described (67) with 30 μg of sheared chromatin and 4 μg anti-Lsd1 antibody (ab17721, Abcam) in a total volume of 240 μL. For histone ChIP, islets were processed as described previously (63); then ChIP was performed as described (68) using 10 or 30 μg of sheared chromatin and 4 μg of anti-H3K4me1 (ab8895, Abcam) or anti-H3K27ac (39133, Active Motif) antibodies, respectively, in a total volume of 240 μL. Lsd1 ChIP of islets from fed or fasted mice was performed as described (69) following nuclear isolation of 1,000 dissociated islets using 30 μL of Dynabeads Protein A beads (Life Technologies) conjugated to 4 μg anti-Lsd1 antibody (ab17721, Abcam) in a total volume of 120 μL. The same protocol was followed for Srf ChIP-Seq in Min6 cells with the following modifications: 1.25 × 10^7^ cells were used for immunoprecipitation using 4 μl of Srf antibody (5147, Cell Signaling Technology) in a total volume of 120 μL. Libraries were constructed from purified DNA using the KAPA DNA Library Preparation Kit for Illumina (Kapa Biosystems). Input libraries were prepared from each replicate using 10 ng of DNA. Libraries were sequenced as above.

### mRNA-Seq data analysis.

Reads were mapped to NCBI37/mm9 (mouse) or GRCh37/hg19 (human) genomes, RPKM was determined, data were filtered, and differentially expressed genes were identified as described (33). Correlations between mRNA-Seq replicates can be found in Supplemental Table 1. GSEA was performed using default settings (70). K-means clustering was performed using R. Enrichment in human gene sets was performed by converting human gene symbols to mouse orthologs using bioDBnet (71).

### ChIP-Seq data analysis.

Reads were mapped to NCBI37/mm9 (mouse) or GRCh37/hg19 (human) genomes using Bowtie2 with a seed length of 33 bp and a maximum of 2 mismatches allowed in the seed region, discarding reads aligning to multiple sites. Duplicate reads were removed using SAMtools. Biological replicates from each condition (*n* = 2 or 3) were assessed for similarity genome-wide using the multiBamSummary program (in “bins” mode) of the deepTools bioinformatics suite (72) (Supplemental Table 1). Human islet H3K27ac ChIP-Seq data and mouse islet TF ChIP-Seq data were downloaded from NCBI’s Gene Expression Omnibus database (GEO GSE30298, GSE40975, GSE54046, GSE62844, GSE70960, GSE72272, GSE84759, GSE126556, GSE132201, and GSE51311) and EMBL-EBI’s ArrayExpress database (E-MTAB-1143 and E-MTAB-1919) and processed in parallel (14, 53, 63, 73–81).

Tag directories were generated from bam files using HOMER (-fragLength 250 option). Replicates were either kept separate (for differential peak calling) or their tag directories were combined using HOMER (for all other analysis). ChIP-Seq peaks within tag directories were originally identified from merged tag directories with the findPeaks command in HOMER (-style histone, -P 0.0000001 for H3K27ac and -style factor for Lsd1) using input tag directories as background. For identification of changes in H3K27ac signal with feeding, all H3K27ac peaks in the fed and fasted states were first merged using BEDtools. The annotatePeaks.pl command in HOMER (-noadj option) was then used to quantify tag densities at these peaks from each biological replicate for subsequent differential peak calling. DEseq2 was used (-norm2total and -batch options) to identify peaks with higher signal (*P* < 0.01) among replicates (*n* = 3) of fed or fasted H3K27ac tag directories. HOMER was used for generating tag densities for genome browser tracks, histograms, heatmaps, and box plots from merged replicates. Tag density box plots (± 1 kb from the center of each peak) were visualized using R. BEDtools was used to determine associations between different coordinate sets (e.g., Lsd1 or H3K27ac peaks) located within ± 1 kb.

Classes of Lsd1-regulated H3K27ac peaks were determined by DEseq2 (with the -norm2total option) using the annotatePeaks.pl command in HOMER (with the -noadj option) for each biological replicate (*n* = 2). Peaks were categorized based on the time point at which they first reached the cutoff of *P* < 0.01 (by DEseq2) in either direction. Further analysis focused on peaks described in Supplemental Figure 5B.

Classes of Lsd1-regulated H3K4me1 patterns at active chromatin were determined using the same analysis as for H3K27ac, resulting in classes of peaks that gain H3K4me1 first at week 1 (342 peaks), peaks that lose H3K4me1 first at week 3 (234 peaks), peaks that never reach this cutoff (37,709 peaks), and a negligible number of peaks that gain H3K4me1 first at week 3 (3 peaks) or lose H3K4me1 first at week 1 (6 peaks).

### Motif enrichment analysis.

Motif enrichments relative to the background set of H3K27ac peaks were determined using HOMER (-size given option).

### Permutation-based significance.

Enrichment tests for associations among transcription start sites (TSSs), Lsd1 peaks, and H3K27ac peaks were determined using a random sampling approach to compare the number of true overlaps to the number of expected overlaps. Null distributions (expected overlap frequencies) were obtained by performing 10,000 iterations of randomly shuffling test coordinates using BEDtools, then intersecting shuffled coordinates with reference coordinates ± 1 kb or ± 10 kb, as indicated, and reporting the percentage of reference sites overlapping with a test site. Permutation data plots are presented with test coordinate sets on the *x* axis and reference coordinate sets as the plot title.

### Gene ontology.

Functional categories from the Kyoto Encyclopedia of Genes and Genomes (KEGG) and Reactome related to the set of feeding-regulated genes and links between each pair of categories were identified with Metascape as previously described (33).

### GWAS enrichment.

Stratified linkage disequilibrium (LD) score regression (82) was used to assess whether LSD1-bound sites were enriched for GWAS signal for metabolic (HOMA-B, ref. 49; HOMA-IR, ref. 49; fasting glucose, ref. 50; fasting glucose adjusted for BMI, ref. 50; fasting insulin, ref. 50; fasting insulin adjusted for BMI, ref. 50; 2-hour glucose adjusted for BMI, ref. 51; fasting proinsulin, ref. 52; and T2D, ref. 48) and well-powered nonmetabolic control traits (downloaded from the GWAS catalog; https://www.ebi.ac.uk/gwas/). Accession numbers were as follows: Alzheimer’s disease, GCST002245; systemic lupus erythematosus, GCST003155; autoimmune vitiligo, GCST004785; primary biliary cirrhosis, GCST003129; primary sclerosing cholangitis, GCST004030; inflammatory bowel disease, GCST004131; rheumatoid arthritis, GCST002318; coronary artery disease, GCST004787; schizophrenia, GCST002539; and major depressive disorder, GCST005839. European subset summary statistics were used where available. After filtering out ENCODE blacklisted regions (23), islet LSD1 peaks were used as a binary annotation. The partitioned heritability version of LD score regression was used to estimate enrichment (%h^2^/%SNPs) using the baseline LD model, version 2.2.

Putative target genes of LSD1-bound sites were determined using promoter capture Hi-C data from primary human islets (17). For each site, gene promoters mapping in a chromatin loop to the site were identified using a 5 kb flanking window around loop anchors. Genetic variants in T2D GWAS data sets were intersected with LSD1-bound sites, retaining variants with at least nominal association (*P* < 0.05) and annotating variants with putative target genes of the overlapping LSD1 site.

### Data availability.

ChIP-Seq and mRNA-Seq data were deposited in the NCBI’s Gene Expression Omnibus database (GEO GSE134901). Accession numbers for human islet H3K27ac ChIP-Seq data used in this study are available in the GEO database (GSE51311) and in the EMBL-EBI’s ArrayExpress database (E-MTAB-1919).

### Statistics.

For islet experiments, islets were pooled from different mice where *n* represents the number of biological replicates using islet pools (i.e., 2 mice per 1 biological replicate, *n* = 1). All in vivo experiments represent individual mice as biological replicates. The exact values of *n* are reported in figure legends. Data are represented as mean ± SEM or as box plots with whiskers spanning data points within the interquartile range × 1.5. Statistical comparisons were performed using Wilcoxon’s rank-sum test, 2-way ANOVA, or unpaired 2-tailed *t* test with Welch’s correction for unequal variance as necessary. Statistical tests were adjusted for multiple comparisons as necessary by the Benjamini-Hochberg procedure or Bonferroni’s correction. Cuffdiff was used to assess expression differences for all pairwise comparisons in mRNA-Seq data, with *P* < 0.01 considered significant. Differential ChIP-Seq peaks were identified using DEseq2 analysis of individual replicates from each group using a cutoff of *P* < 0.01. For GSEA, significance was assessed with 1,000 permutations and FDR was used to correct for multiple testing. All other statistical analysis was performed in R.

### Study approval.

All animal experiments were approved by the Institutional Animal Care and Use Committee of UCSD.

## Author contributions

M Wortham and MS conceived the study, were responsible for its overall design, and prepared the manuscript. M Wortham, FL, and JYF performed mouse experiments. FL performed islet isolations. M Wortham, JYF, ARH, BRC, NAP, YJ, and MOH performed islet experiments and hormone measurements. M Wortham, NKV, NAP, and USJ generated ChIP-Seq data. M Wortham, M Wallace, and CMM performed and/or interpreted glucose-tracing experiments. M Wortham, FM, MM, GW, NKV, and YS performed bioinformatics analysis. JC, CM, and KJG analyzed human genetic data. RS provided key reagents. OSS designed and interpreted islet respirometry experiments.

## Supplementary Material

Supplemental data

Supplemental table 2

Supplemental table 3

Supplemental table 4

Supplemental table 5

## Version 1 02/23/2023

In-Press Preview

## Version 2 04/17/2023

Electronic publication

## References

[1] Cahill GF, (2006). Fuel metabolism in starvation. Annu Rev Nutr.

[2] Capozzi ME (2019). β Cell tone is defined by proglucagon peptides through cAMP signaling. JCI Insight.

[3] Prentki M (2013). Metabolic signaling in fuel-induced insulin secretion. Cell Metab.

[4] Henquin JC (2000). Triggering and amplifying pathways of regulation of insulin secretion by glucose. Diabetes.

[5] Bjorkman O, Eriksson LS (1985). Influence of a 60-hour fast on insulin-mediated splanchnic and peripheral glucose metabolism in humans. J Clin Invest.

[6] Burch PT (1981). Adaptation of glycolytic enzymes: glucose use and insulin release in rat pancreatic islets during fasting and refeeding. Diabetes.

[7] Zhou YP (1999). Basal insulin hypersecretion in insulin-resistant Zucker diabetic and Zucker fatty rats: role of enhanced fuel metabolism. Metabolism.

[8] Wortham M, Sander M (2016). Mechanisms of β-cell functional adaptation to changes in workload. Diabetes Obes Metab.

[9] Gremlich S (2005). Pancreatic islet adaptation to fasting is dependent on peroxisome proliferator-activated receptor alpha transcriptional up-regulation of fatty acid oxidation. Endocrinology.

[10] Glauser DA (2007). Transcriptional response of pancreatic beta cells to metabolic stimulation: large scale identification of immediate-early and secondary response genes. BMC Mol Biol.

[11] Jhala US (2003). cAMP promotes pancreatic beta-cell survival via CREB-mediated induction of IRS2. Genes Dev.

[12] Miranda JG (2022). Dynamic changes in β-cell [Ca^2+^] regulate NFAT activation, gene transcription, and islet gap junction communication. Mol Metab.

[13] Schmidt SF (2016). Integrative genomics outlines a biphasic glucose response and a ChREBP-RORγ axis regulating proliferation in β cells. Cell Rep.

[14] Van de Velde S (2019). CREB promotes beta cell gene expression by targeting its coactivators to tissue-specific enhancers. Mol Cell Biol.

[15] Cluntun AA (2015). The rate of glycolysis quantitatively mediates specific histone acetylation sites. Cancer Metab.

[16] Goldstein I (2017). Transcription factor assisted loading and enhancer dynamics dictate the hepatic fasting response. Genome Res.

[17] Miguel-Escalada I (2019). Human pancreatic islet three-dimensional chromatin architecture provides insights into the genetics of type 2 diabetes. Nat Genet.

[18] Cao K (2018). An Mll4/COMPASS-Lsd1 epigenetic axis governs enhancer function and pluripotency transition in embryonic stem cells. Sci Adv.

[19] Whyte WA (2012). Enhancer decommissioning by LSD1 during embryonic stem cell differentiation. Nature.

[20] Hino S (2012). FAD-dependent lysine-specific demethylase-1 regulates cellular energy expenditure. Nat Commun.

[21] Duteil D (2014). LSD1 promotes oxidative metabolism of white adipose tissue. Nat Commun.

[22] Hatori M (2012). Time-restricted feeding without reducing caloric intake prevents metabolic diseases in mice fed a high-fat diet. Cell Metab.

[23] ENCODE Project Consortium (2012). An integrated encyclopedia of DNA elements in the human genome. Nature.

[24] Kim-Muller JY (2016). FoxO1 deacetylation decreases fatty acid oxidation in β-cells and sustains insulin secretion in diabetes. J Biol Chem.

[25] Wei Y (2015). The stress-responsive kinases MAPKAPK2/MAPKAPK3 activate starvation-induced autophagy through Beclin 1 phosphorylation. Elife.

[26] Piccand J (2014). Pak3 promotes cell cycle exit and differentiation of β-cells in the embryonic pancreas and is necessary to maintain glucose homeostasis in adult mice. Diabetes.

[27] Hnisz D (2013). Super-enhancers in the control of cell identity and disease. Cell.

[28] Blanchet E (2015). Feedback inhibition of CREB signaling promotes beta cell dysfunction in insulin resistance. Cell Rep.

[29] Kim-Muller JY (2014). Metabolic inflexibility impairs insulin secretion and results in MODY-like diabetes in triple FoxO-deficient mice. Cell Metab.

[30] Chen B (2018). Auto-fatty acylation of transcription factor RFX3 regulates ciliogenesis. Proc Natl Acad Sci U S A.

[31] Gualdrini F (2016). SRF co-factors control the balance between cell proliferation and contractility. Mol Cell.

[32] Duteil D (2016). Lsd1 ablation triggers metabolic reprogramming of brown adipose tissue. Cell Rep.

[33] Wortham M (2018). Integrated in vivo quantitative proteomics and nutrient tracing reveals age-related metabolic rewiring of pancreatic β cell function. Cell Rep.

[34] Farfari S (2000). Glucose-regulated anaplerosis and cataplerosis in pancreatic beta-cells: possible implication of a pyruvate/citrate shuttle in insulin secretion. Diabetes.

[35] Shi Y (2004). Histone demethylation mediated by the nuclear amine oxidase homolog LSD1. Cell.

[36] Lee YS (2013). The fractalkine/CX3CR1 system regulates β cell function and insulin secretion. Cell.

[37] Kalwat MA (2013). A p21-activated kinase (PAK1) signaling cascade coordinately regulates F-actin remodeling and insulin granule exocytosis in pancreatic β cells. Biochem Pharmacol.

[38] Zmuda EJ (2010). The roles of ATF3, an adaptive-response gene, in high-fat-diet-induced diabetes and pancreatic beta-cell dysfunction. Mol Endocrinol.

[39] Reynolds MS (2016). β-cell deletion of Nr4a1 and Nr4a3 nuclear receptors impedes mitochondrial respiration and insulin secretion. Am J Physiol Endocrinol Metab.

[40] Ray JD (2016). Nkx6.1-mediated insulin secretion and β-cell proliferation is dependent on upregulation of c-Fos. FEBS Lett.

[41] Schuit F (1999). Cellular origin of hexokinase in pancreatic islets. J Biol Chem.

[42] Epstein PN (1992). Expression of yeast hexokinase in pancreatic beta cells of transgenic mice reduces blood glucose, enhances insulin secretion, and decreases diabetes. Proc Natl Acad Sci U S A.

[43] Thorrez L (2011). Tissue-specific disallowance of housekeeping genes: the other face of cell differentiation. Genome Res.

[44] Pullen TJ (2017). Analysis of purified pancreatic islet beta and alpha cell transcriptomes reveals 11β-hydroxysteroid dehydrogenase (Hsd11b1) as a novel disallowed gene. Front Genet.

[45] Lemaire K (2016). Disallowed and allowed gene expression: two faces of mature islet beta cells. Annu Rev Nutr.

[46] Kuo T (2019). Induction of α cell-restricted Gc in dedifferentiating β cells contributes to stress-induced β cell dysfunction. JCI Insight.

[47] Zeng C (2017). Pseudotemporal ordering of single cells reveals metabolic control of postnatal β cell proliferation. Cell Metab.

[48] Mahajan A (2018). Fine-mapping type 2 diabetes loci to single-variant resolution using high-density imputation and islet-specific epigenome maps. Nat Genet.

[49] Dupuis J (2010). New genetic loci implicated in fasting glucose homeostasis and their impact on type 2 diabetes risk. Nat Genet.

[50] Manning AK (2012). A genome-wide approach accounting for body mass index identifies genetic variants influencing fasting glycemic traits and insulin resistance. Nat Genet.

[51] Saxena R (2010). Genetic variation in GIPR influences the glucose and insulin responses to an oral glucose challenge. Nat Genet.

[52] Strawbridge RJ (2011). Genome-wide association identifies nine common variants associated with fasting proinsulin levels and provides new insights into the pathophysiology of type 2 diabetes. Diabetes.

[53] Pasquali L (2014). Pancreatic islet enhancer clusters enriched in type 2 diabetes risk-associated variants. Nat Genet.

[54] Lee MG (2006). Functional interplay between histone demethylase and deacetylase enzymes. Mol Cell Biol.

[55] Song Y (2020). Mechanism of crosstalk between the LSD1demethylase and HDAC1 deacetylase in the CoREST complex. Cell Rep.

[56] Hsieh WC (2022). Glucose starvation induces a switch in the histone acetylome for activation of gluconeogenic and fat metabolism genes. Mol Cell.

[57] Drong AW (2012). The genetic and epigenetic basis of type 2 diabetes and obesity. Clin Pharmacol Ther.

[58] Wang J (2007). Opposing LSD1 complexes function in developmental gene activation and repression programmes. Nature.

[59] Gu G (2002). Direct evidence for the pancreatic lineage: NGN3+ cells are islet progenitors and are distinct from duct progenitors. Development.

[60] Tamarina NA (2014). Characterization of mice expressing Ins1 gene promoter driven CreERT recombinase for conditional gene deletion in pancreatic β-cells. Islets.

[61] Benner C (2014). The transcriptional landscape of mouse beta cells compared to human beta cells reveals notable species differences in long non-coding RNA and protein-coding gene expression. BMC Genomics.

[62] Ramms B (2022). Systemic LSD1 inhibition prevents aberrant remodeling of metabolism in obesity. Diabetes.

[63] Taylor BL (2013). Nkx6.1 is essential for maintaining the functional state of pancreatic beta cells. Cell Rep.

[64] Chailangkarn T (2016). A human neurodevelopmental model for Williams syndrome. Nature.

[65] Bramswig NC (2013). Epigenomic plasticity enables human pancreatic α to β cell reprogramming. J Clin Invest.

[66] Xie R (2013). Dynamic chromatin remodeling mediated by polycomb proteins orchestrates pancreatic differentiation of human embryonic stem cells. Cell Stem Cell.

[67] Vinckier NK (2020). LSD1-mediated enhancer silencing attenuates retinoic acid signalling during pancreatic endocrine cell development. Nat Commun.

[68] Ibarra Urizar A (2019). Beta-cell dysfunction induced by non-cytotoxic concentrations of Interleukin-1beta is associated with changes in expression of beta-cell maturity genes and associated histone modifications. Mol Cell Endocrinol.

[69] Seidman JS (2020). Niche-specific reprogramming of epigenetic landscapes drives myeloid cell diversity in nonalcoholic steatohepatitis. Immunity.

[70] Subramanian A (2005). Gene set enrichment analysis: a knowledge-based approach for interpreting genome-wide expression profiles. Proc Natl Acad Sci U S A.

[71] Mudunuri U (2009). bioDBnet: the biological database network. Bioinformatics.

[72] Ramirez F (2016). deepTools2: a next generation web server for deep-sequencing data analysis. Nucleic Acids Res.

[73] Parker SC (2013). Chromatin stretch enhancer states drive cell-specific gene regulation and harbor human disease risk variants. Proc Natl Acad Sci U S A.

[74] Ediger BN (2017). LIM domain-binding 1 maintains the terminally differentiated state of pancreatic β cells. J Clin Invest.

[75] Ait-Lounis A (2010). The transcription factor Rfx3 regulates beta-cell differentiation, function, and glucokinase expression. Diabetes.

[76] Piccand J (2014). Rfx6 maintains the functional identity of adult pancreatic β cells. Cell Rep.

[77] Tennant BR (2013). Identification and analysis of murine pancreatic islet enhancers. Diabetologia.

[78] Jia S (2015). Insm1 cooperates with Neurod1 and Foxa2 to maintain mature pancreatic β-cell function. EMBO J.

[79] Kuo T (2019). Identification of *C2CD4A* as a human diabetes susceptibility gene with a role in β cell insulin secretion. Proc Natl Acad Sci U S A.

[80] Perelis M (2015). Pancreatic β cell enhancers regulate rhythmic transcription of genes controlling insulin secretion. Science.

[81] Khoo C (2012). Research resource: the pdx1 cistrome of pancreatic islets. Mol Endocrinol.

[82] Bulik-Sullivan BK (2015). LD score regression distinguishes confounding from polygenicity in genome-wide association studies. Nat Genet.

