# Supplementary material for: Nutrient regulation of the islet epigenome controls adaptive insulin secretion
Source: J Clin Invest. 2023 Apr 17;133(8):e165208. doi: 10.1172/JCI165208 (PMC10104905; doi:10.1172/JCI165208)
Supplement: Supplemental data [file jci-133-165208-s007.pdf]

Supplemental Information

Supplemental Figures

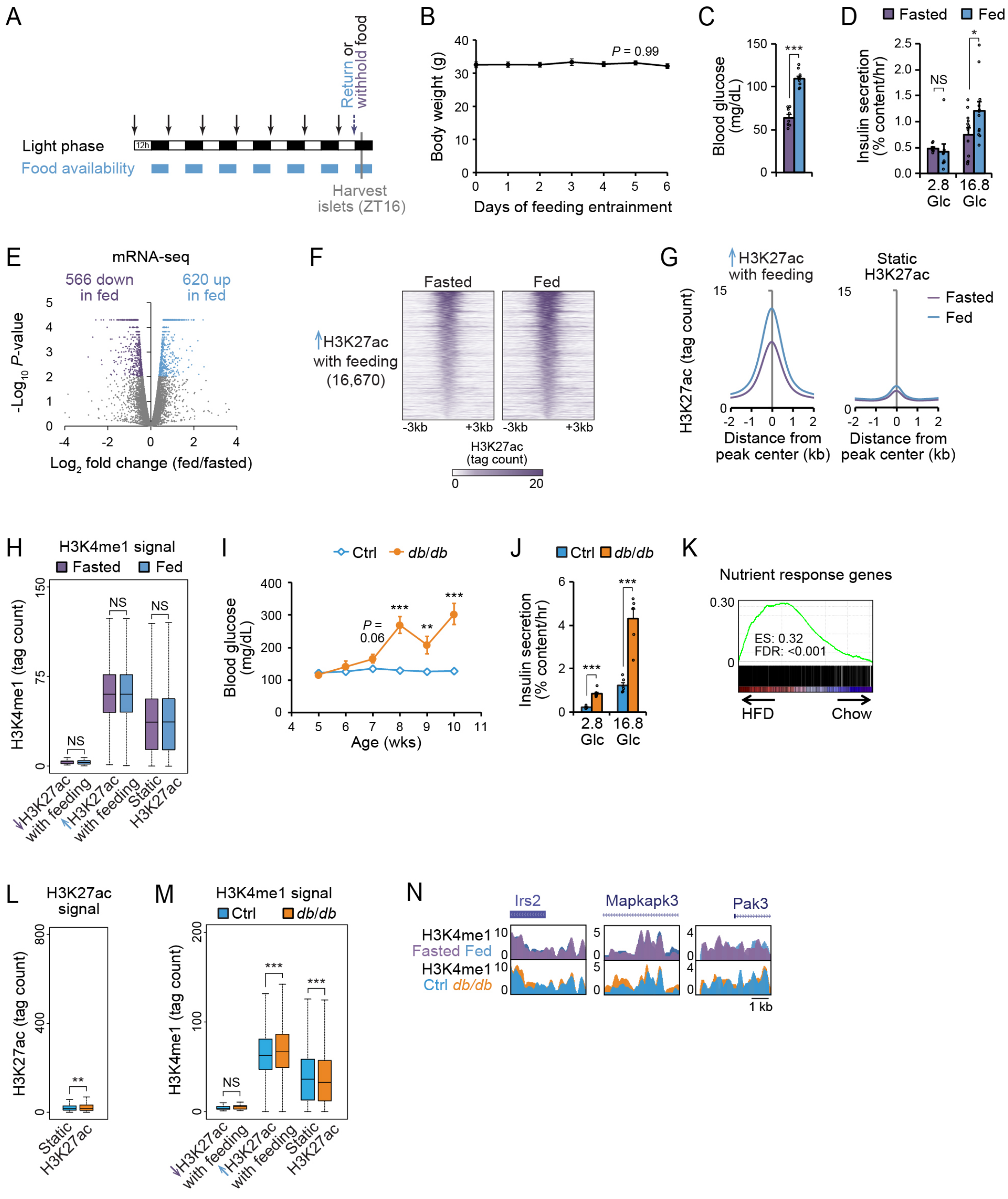

Supplemental Figure 1. Short-term and prolonged increases in nutrient intake enhance glucose stimulated insulin secretion.
(A) Schematic of time-restricted feeding experiment used to entrain food intake in mice. Arrows indicate food removal and body weight measurements. ZT, Zeitgeber time; h, hours.
(B) Animal weights at the indicated days of time-restricted feeding (taken at ZT0). *n* = 20 mice. *P*-value was calculated by one-way ANOVA.

(C) Blood glucose levels at the time of islet isolation (ZT16). Fasted: *n* = 8 mice, fed: *n* = 9 mice. ****P* < 0.001 by unpaired two-tailed t-test.
(D) Static insulin secretion assay in islets from fed and fasted mice stimulated with the indicated glucose (Glc) concentrations (in mM). Fasted or fed 2.8 mM glucose: *n* = 8 pools of 10 islets each, fasted or fed 16.8 mM glucose: *n* = 12 islet pools. **P* < 0.05, by unpaired two-tailed t-test with Welch's correction for unequal variance as necessary.
(E) Volcano plot comparing mRNA levels in islets from fed and fasted mice. Differentially expressed genes are indicated in purple or blue (*P* < 0.01 by Cuffdiff). *n* = 3 biological replicates of islets pooled from separate mice per group.
(F and **G)** Heatmaps (F) and histograms (G) of H3K27ac ChIP-seq signal for the indicated classes of H3K27ac peaks (*P* < 0.01 by DEseq2). *n* = 3 biological replicates of islets pooled from separate mice per group; data from independent ChIP-seq experiments were merged to generate heatmaps and histograms.
(H) ChIP-seq signal for H3K4me1 at the indicated classes of H3K27ac peaks in islets from fed and fasted mice. *n* = 2 biological replicates of islets pooled from separate mice per group; data from independent ChIP-seq experiments were subsequently merged to generate boxplots. Boxplot whiskers span data points within the interquartile range x 1.5. NS, not significant.
(I) Time course of ad libitum-fed blood glucose levels in control (ctrl, *db*/+; *n* = 19) and *db*/*db* (*n* = 18) mice at the indicated ages in weeks (wks). Data are shown as mean ± S.E.M. ***P* < 0.01, ****P* < 0.001 by unpaired two-tailed t-test with Welch's correction for unequal variance as necessary.
(J) Static insulin secretion assay in 10-week-old control and *db*/*db* islets stimulated with the indicated glucose (Glc) concentrations (in mM). Control 2.8 mM glucose: *n* = 4 pools of 10 islets each, all other groups: *n* = 6 islet pools. Data in are shown as mean ± S.E.M. ****P* < 0.001 by unpaired two-tailed t-test with Welch's correction for unequal variance.
(K) Gene set enrichment analysis of nutrient response genes (from Figure 1I) against islet mRNA-seq data from mice fed a high-fat diet (HFD) for 27 weeks (1).
(L and **M)** ChIP-seq signal for H3K27ac (L) and H3K4me1 (M) at the indicated classes of H3K27ac peaks in islets from *db/db* and control (ctrl, *db*/+) islets. *n* = 2 biological replicates of islets pooled from separate mice per group; data from independent ChIP-seq experiments were subsequently merged to generate boxplots. Boxplot whiskers span data points within the interquartile range x 1.5. ***P* < 0.01, ****P* < 0.001 by Wilcoxon rank-sum test; NS, not significant.
(N) H3K4me1 ChIP-seq genome browser tracks for genes shown in Figure 1K in islets from fed and fasted mice and in *db/db* and control islets.
Data are shown as mean ± S.E.M. unless otherwise indicated.

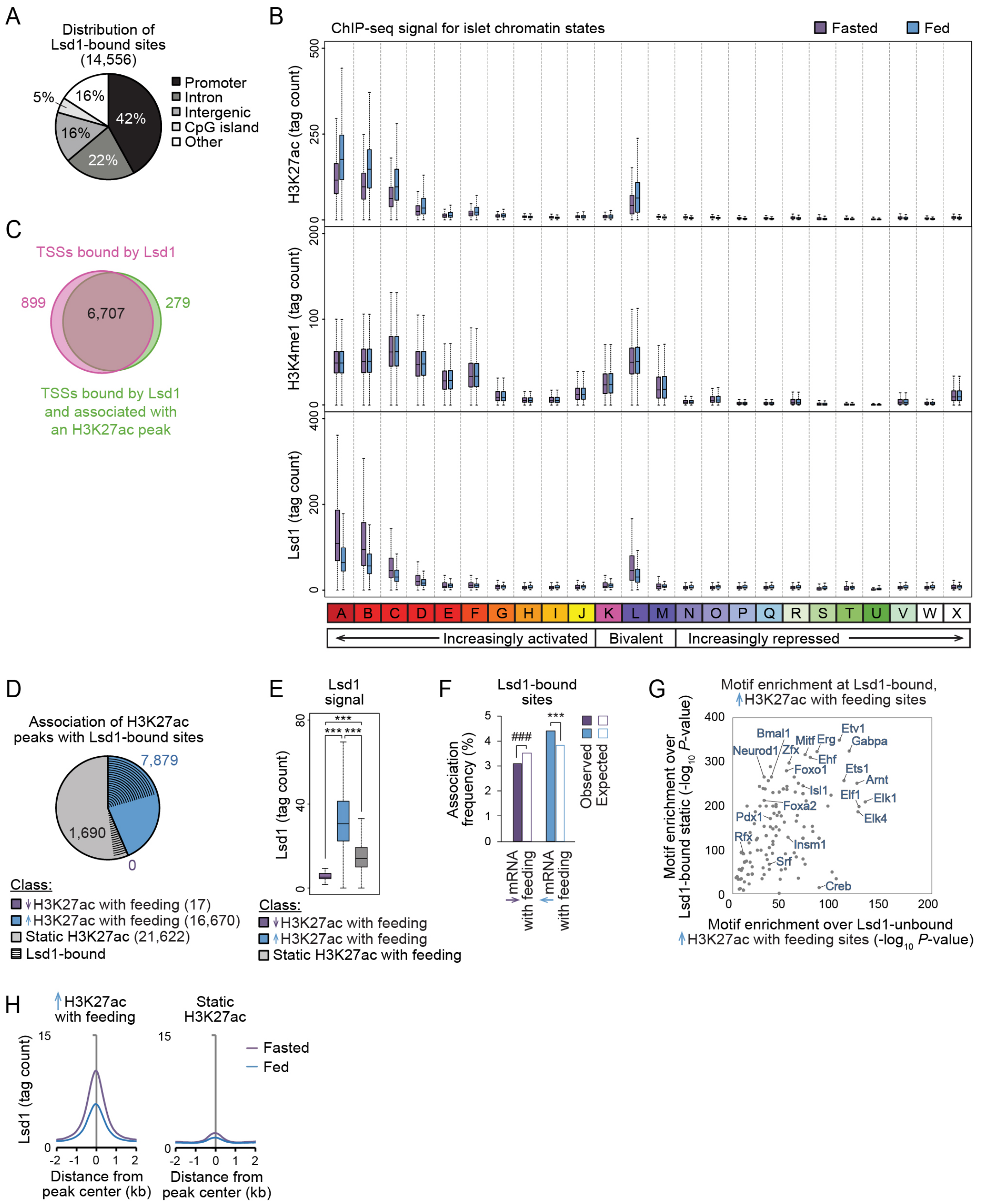

Supplemental Figure 2. Lsd1 associates with feeding-regulated active chromatin.
(A) Distribution of Lsd1 peaks in islets from ad libitum-fed mice at the indicated genomic features. Lsd1 ChIP-seq data are from *n* = 1 biological replicate from pooled islets. Data were highly correlated with Lsd1 ChIP-seq data from an independent biological replicate (see Supplemental Table 1).
(B) ChIP-seq signal for H3K27ac, H3K4me1, and Lsd1 at the indicated chromatin states from (2). *n* = 3 (H3K27ac) or *n* = 2 (H3K4me1 and Lsd1) biological replicates of islets pooled from separate mice per group; data from independent ChIP-seq experiments were subsequently merged to generate boxplots. Boxplot whiskers span data points within the interquartile range x 1.5.

(C) Venn diagram comparing the number of transcription start sites (TSSs) associated ± 50 kb with an Lsd1 peak and TSSs associated ± 50 kb with an H3K27ac peak within ± 1 kb of an Lsd1 peak.
(D) Proportion of the indicated classes of H3K27ac peaks associating with an Lsd1 peak ± 1 kb. Numbers indicate H3K27ac peaks associated with an Lsd1 peak.
(E) Lsd1 ChIP-seq signal at the indicated classes of H3K27ac peaks. Lsd1 ChIP-seq data are from *n* = 1 biological replicate from pooled islets of ad libitum-fed mice. Data were highly correlated with Lsd1 ChIP-seq data from an independent biological replicate (see Supplemental Table 1).
(F) Association frequencies between TSSs of genes downregulated or upregulated by feeding with an Lsd1 peak ± 10 kb. Open bars indicate association frequencies expected by chance. $^{**}P$ < 0.01 for enrichment and $^{###}$P < 0.001 for depletion by permutation test.
(G) Enrichment of transcription factor (TF) motifs for the indicated TFs at H3K27ac peaks gaining acetylation with feeding and associated with an Lsd1 peak ± 1 kb relative to static H3K27ac peaks associated with an Lsd1 peak ± 1 kb plotted against enrichment of motifs at the same sites relative to H3K27ac peaks gaining acetylation with feeding and not associated with an Lsd1 peak.
(H) Histograms of Lsd1 ChIP-seq signal for the indicated classes of H3K27ac peaks. *n* = 2 biological replicates of islets pooled from separate mice per group; data from independent ChIP-seq experiments were merged to generate histograms.

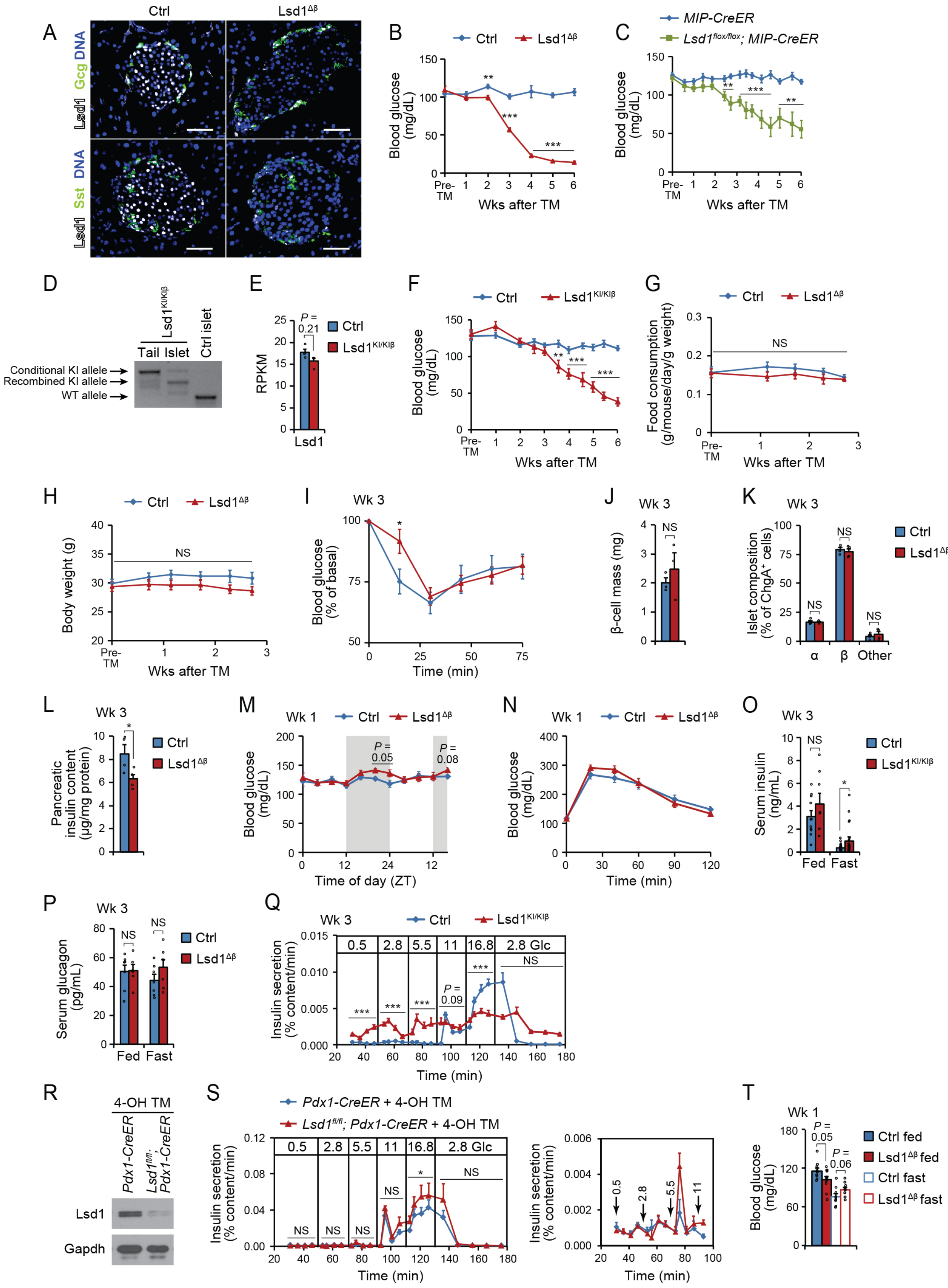
A
Ctrl
Lsd1Δβ
Lsd1 Gcg DNA
Lsd1 Sst DNA
B
Ctrl
Lsd1Δβ
Blood glucose (mg/dL)
Wks after TM
Pre-TM
C
MIP-CreER
Lsd1flox/flox; MIP-CreER
Blood glucose (mg/dL)
Wks after TM
D
Lsd1KI/KIβ
Tail
Islet
Ctrl islet
Conditional KI allele
Recombined KI allele
WT allele
E
P = 0.21
Ctrl
Lsd1KI/KIβ
RPKM
Lsd1
F
Ctrl
Lsd1KI/KIβ
Blood glucose (mg/dL)
Wks after TM
G
Ctrl
Lsd1Δβ
Food consumption (g/mouse/day/g weight)
NS
Wks after TM
H
Ctrl
Lsd1Δβ
Body weight (g)
NS
Wks after TM
I
Wk 3
Blood glucose (% of basal)
Time (min)
J
Wk 3
NS
β-cell mass (mg)
K
Wk 3
Ctrl
Lsd1Δβ
Islet composition (% of ChgA+ cells)
α
β
Other
L
Wk 3
Ctrl
Lsd1Δβ
Pancreatic insulin content (μg/mg protein)
M
Wk 1
Ctrl
Lsd1Δβ
P = 0.05
P = 0.08
Blood glucose (mg/dL)
Time of day (ZT)
N
Wk 1
Ctrl
Lsd1Δβ
Blood glucose (mg/dL)
Time (min)
O
Wk 3
Ctrl
Lsd1KI/KIβ
Serum insulin (ng/mL)
Fed
Fast
P
Wk 3
Ctrl
Lsd1Δβ
Serum glucagon (pg/mL)
Fed
Fast
Q
Wk 3
Ctrl
Lsd1KI/KIβ
0.5
2.8
5.5
11
16.8
2.8 Glc
P = 0.09
Insulin secretion (% content/min)
Time (min)
R
4-OH TM
Pdx1-CreER
Lsd1fl/fl; Pdx1-CreER
Lsd1
Gapdh
S
Pdx1-CreER + 4-OH TM
Lsd1fl/fl; Pdx1-CreER + 4-OH TM
0.5
2.8
5.5
11
16.8
2.8 Glc
Insulin secretion (% content/min)
Time (min)
T
Wk 1
P = 0.05
P = 0.06
Ctrl fed
Lsd1Δβ fed
Ctrl fast
Lsd1Δβ fast
Blood glucose (mg/dL)

Supplemental Figure 3. Hypoglycemia in Lsd1$^{\Delta\beta}$ mice is not caused by altered insulin sensitivity, food intake, β-cell mass, or circulating glucagon.

(A) Immunofluorescence staining for the indicated proteins on pancreas sections from control (ctrl, TM-treated *Lsd1$^{flox/+}$*; *Pdx1-CreER* mice) and Lsd1$^{\Delta\beta}$ mice two days after TM treatment. Scale bar, 50 μm.

(B) Time course of ad libitum-fed blood glucose levels in female control (TM-treated *Lsd1$^{+/+}$*; *Pdx1-CreER* mice, *n* = 11) and Lsd1$^{\Delta\beta}$ (*n* = 10) mice. pre-TM, within 3 days prior to initial TM injection.

(C) Time course of ad libitum-fed blood glucose levels in *Lsd1$^{+/+}$*; *MIP-CreER* (*n* = 12) and *Lsd1$^{flox/flox}$; MIP-CreER* (*n* = 8) mice following TM treatment.

(D) PCR of genomic DNA from the indicated tissues using primers detecting recombination of the catalytically inactive *Lsd1* knock-in (KI) allele from control (ctrl, TM-treated *Lsd1$^{+/+}$*; *Pdx1-CreER* mice) or Lsd1$^{KI/KI\beta}$ (TM-treated *Lsd1$^{KI/KI}$*; *Pdx1-CreER*) mice two days after TM treatment.

(E) *Lsd1* mRNA level from mRNA-seq in the indicated groups of islets three weeks following TM administration. *n* = 4 biological replicates of islets pooled from separate mice per group. *P*-value from Cuffdiff is indicated.

(F) Time course of ad libitum-fed blood glucose levels in control (*n* = 13) and Lsd1$^{KI/KI\beta}$ (*n* = 11) mice.

(G and **H)** Time courses of food intake (G) and body weight (H) in control and Lsd1$^{\Delta\beta}$ mice following TM treatment. *n* = 5 cages of mice per group (G), *n* = 10 mice per group (H).

(I) Insulin tolerance tests in control (*n* = 7) and Lsd1$^{\Delta\beta}$ (*n* = 9) mice three weeks following TM treatment.

(J-L) β-cell mass (J), islet cell type composition (K), and pancreatic insulin content (L) of control and Lsd1$^{\Delta\beta}$ mice three weeks following TM treatment. *n* = 3 (J) or 4 (K, L) mice per group.

(M) Blood glucose levels in control and Lsd1$^{\Delta\beta}$ mice at 4-hour increments across a 40-hour time course in control and Lsd1$^{\Delta\beta}$ mice one week following TM treatment. *n* = 7 mice per group.

(N) Glucose tolerance tests in control (*n* = 12) and Lsd1$^{\Delta\beta}$ (*n* = 15) mice one week following TM treatment.

(O) Serum insulin levels in ad libitum-fed and 16-hour fasted mice of the indicated genotypes three weeks following TM treatment. *n* = 11 fed ctrl mice, *n* = 8 fed Lsd1$^{KI/KI\beta}$ mice, *n* = 23 fasted ctrl mice, and *n* = 25 fasted Lsd1$^{KI/KI\beta}$ mice.

(P) Serum glucagon levels in ad libitum-fed and 16-hour fasted mice of the indicated genotypes three weeks following TM treatment. Fed Lsd1$^{\Delta\beta}$: *n* = 6, all other groups: *n* = 7 mice per group.

(Q) Insulin secretion by control and Lsd1$^{KI/KI\beta}$ islets during perifusion with the indicated glucose (Glc) concentrations (in mM) three weeks following TM treatment. *n* = 4 pools of 130 islets each. ****P* < 0.001 by two-way ANOVA for genotype for each time block.

(R) Western blot for the indicated proteins 72 hours following treatment of the indicated genotypes of islets with 2 μM 4-OH TM (4-hydroxy-tamoxifen).

(S) Insulin secretion by the indicated genotypes of islets during perifusion with the indicated glucose (Glc) concentrations (in mM) 96 hours following treatment with 2 μM 4-OH TM. Below, data shown at a reduced scale for the indicated time points. *n* = 4 pools of 130 islets each. **P* < 0.05 by two-way ANOVA for genotype for each time block.

(T) Blood glucose levels of the indicated mice that were feeding-entrained and then fed or fasted as in Supplemental Figure 1A. *n* = 9 fasted ctrl mice, *n* = 10 fed ctrl and fasted Lsd1$^{\Delta\beta}$ mice, *n* = 11 fed Lsd1$^{\Delta\beta}$ mice.

Wk/s, week/s; TM, tamoxifen; fl, flox. Data are shown as mean ± S.E.M. **P* < 0.05, ***P* < 0.01, ****P* < 0.001 by unpaired two-tailed t-test with Welch's correction for unequal variance as necessary (B, C, F-P, T). NS, not significant.

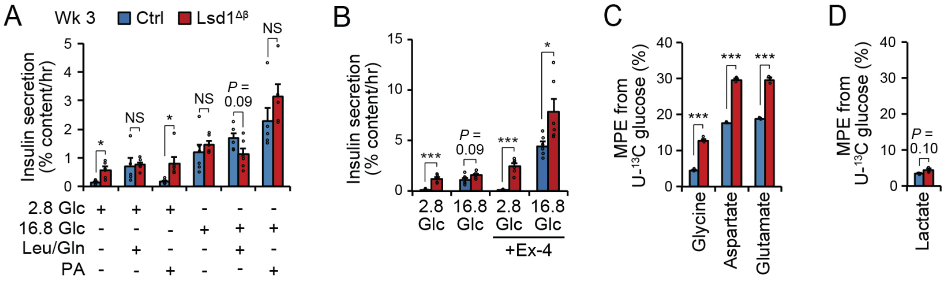

Supplemental Figure 4. Functional and metabolic characteristics of islets from Lsd1$^{\Delta\beta}$ mice.

(A) Static insulin secretion assays in control (ctrl) and Lsd1$^{\Delta\beta}$ islets stimulated with the indicated glucose (Glc) concentrations (in mM) with or without amino acids leucine and glutamine (Leu/Gln) or the fatty acid palmitate (PA). Control 2.8 mM Glucose + Leu/Gln, Control 2.8 mM Glucose + PA, Control 16.8 mM Glucose + Leu/Gln, Control 16.8 mM Glucose + PA, Lsd1$^{\Delta\beta}$ 16.8 mM Glucose + PA: *n* = 5 pools of 10 islets each per group, all other groups: *n* = 6 islet pools.

(B) Static insulin secretion assay in control and Lsd1$^{\Delta\beta}$ islets stimulated with the indicated glucose (Glc) concentrations (in mM) with and without Exendin-4 (Ex-4) three weeks following TM treatment. Control 2.8 mM glucose: *n* = 4 pools of 10 islets each, control 2.8 mM glucose + Ex-4: *n* = 5 islet pools, all other groups: *n* = 6 islet pools. *$P < 0.05$, ***$P < 0.001$ by unpaired two-tailed t-test with Welch's correction for unequal variance as necessary.

(C and **D)** Molar percent enrichment (MPE) of ^{13}C for amino acids (C) and lactate (D) in islets following two hours of tracing in 2.8 mM U-^{13}C glucose. *n* = 3 pools of 220 islets each.

Islets were isolated from control and Lsd1$^{\Delta\beta}$ animals three weeks following tamoxifen treatment for all experiments. Data are shown as mean ± S.E.M. *$P < 0.05$, ***$P < 0.001$ by unpaired two-tailed t-test with Welch's correction for unequal variance as necessary. NS, not significant.

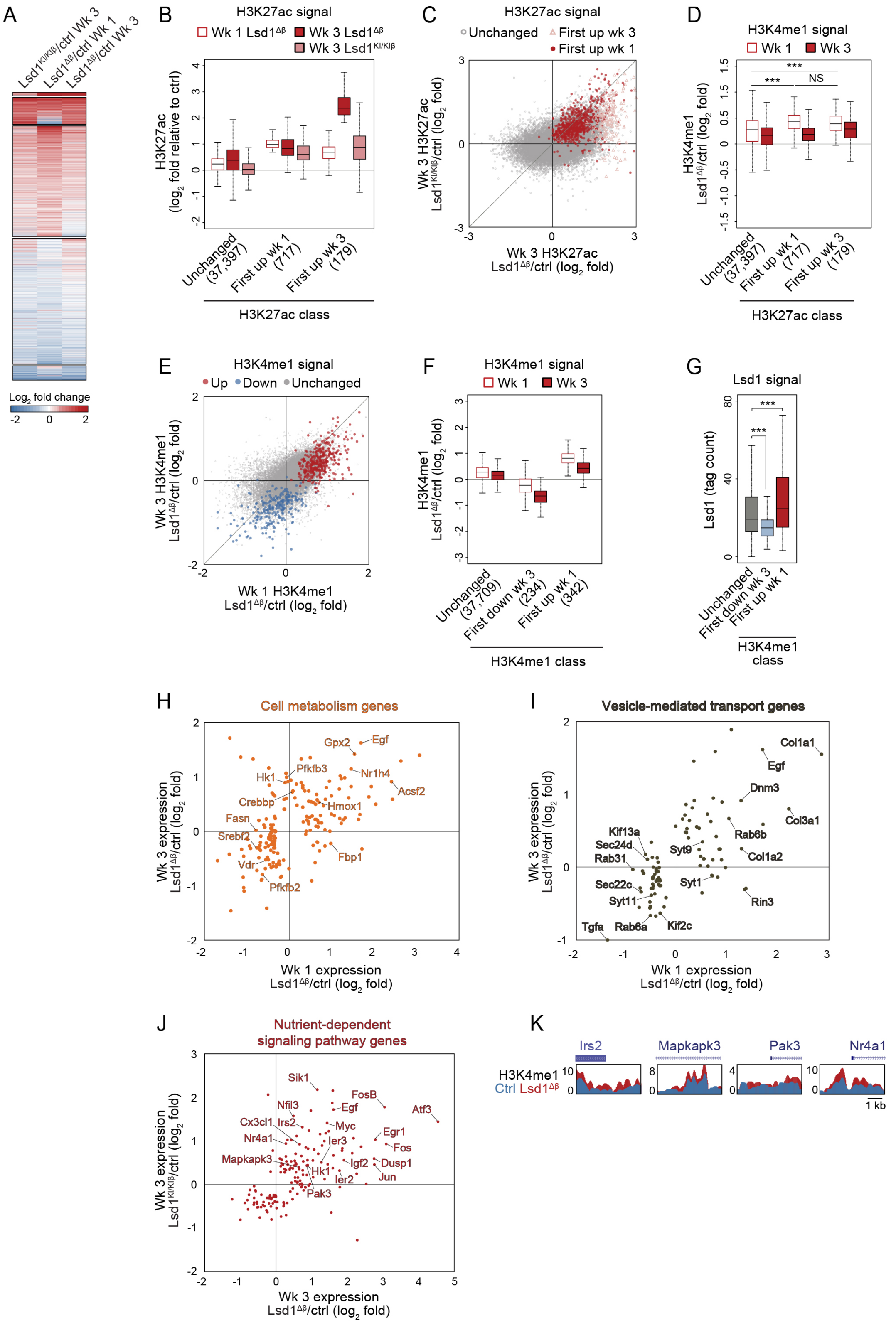

Supplemental Figure 5. Concordant changes in H3K27ac and H3K4me1 after *Lsd1* inactivation.

(A) K-means clustering of $\log_2$ fold changes in mRNA levels in $Lsd1^{\Delta\beta}$ islets or $Lsd1^{KI/KI\beta}$ islets compared to respective control islets for the indicated time points following TM treatment for all differentially expressed genes ($P < 0.01$ by Cuffdiff). $Lsd1^{\Delta\beta}$ week three: $n = 5$ biological replicates of islets pooled from separate mice per group, $Lsd1^{KI/KI\beta}$ and respective controls: $n = 4$ biological replicates, all other groups: $n = 3$ biological replicates. Wk, week.
(B) ChIP-seq signal for H3K27ac at the indicated H3K27ac peaks classified by changes in $Lsd1^{\Delta\beta}$ islets (from Figure 5C) plotted as relative tag density in $Lsd1^{\Delta\beta}$ or $Lsd1^{KI/KI\beta}$ islets compared to control (ctrl) islets at the indicated time points following TM treatment. $Lsd1^{KI/KI\beta}$ islets: $n = 3$ biological replicates. All other groups: $n = 2$ biological replicates of islets pooled from separate mice per group. Data from independent ChIP-seq experiments were merged for analysis. Boxplot whiskers span data points within the interquartile range x 1.5.
(C) H3K27ac ChIP-seq signal at the indicated classes of H3K27ac peaks (as in B) plotted as relative tag density in $Lsd1^{\Delta\beta}$ or $Lsd1^{KI/KI\beta}$ islets compared to respective control islets three weeks following TM treatment.
(D) ChIP-seq signal for H3K4me1 at the indicated classes of H3K27ac peaks (from Figure 5C) plotted as relative tag density in $Lsd1^{\Delta\beta}$ islets compared to control (ctrl) islets at the indicated time points following TM treatment. $n = 2$ biological replicates of islets pooled from separate mice per group; data from independent ChIP-seq experiments were subsequently merged for analysis. Boxplot whiskers span data points within the interquartile range x 1.5.
(E) H3K4me1 ChIP-seq signal at H3K27ac peaks (from Supplemental Figure 2D) plotted as relative tag density in $Lsd1^{\Delta\beta}$ islets compared to control islets at the indicated time points following TM treatment. The gray line indicating a slope of 1 provides a reference for corresponding changes at the two time points. Peaks exhibiting significantly different tag densities ($P < 0.01$ by DEseq2) are indicated by color. $n = 2$ biological replicates of islets pooled from separate mice per group; data from independent ChIP-seq experiments were subsequently merged to generate the scatterplot.
(F) H3K4me1 ChIP-seq signal at H3K27ac peaks plotted as relative tag density in $Lsd1^{\Delta\beta}$ islets compared to control (ctrl) islets at one and three weeks (Wk) following TM treatment. Peaks were classified by the time point at which differences in H3K4me1 tag density between control and $Lsd1^{\Delta\beta}$ islets first reached statistical significance ($P < 0.01$ by DEseq2; see Methods for additional details on peak classification). $n = 2$ biological replicates of islets pooled from separate mice per group; data from independent ChIP-seq experiments were subsequently merged to generate boxplots. Boxplot whiskers span data points within the interquartile range x 1.5.
(G) Lsd1 ChIP-seq signal at the indicated classes of H3K4me1 sites (from E). Lsd1 ChIP-seq data are from $n = 1$ biological replicate from pooled islets. Data were validated to be highly correlated with ChIP-seq data prepared from an independent biological replicate. Boxplot whiskers span data points within the interquartile range x 1.5.
(H-J) Log_2 fold changes in mRNA levels between $Lsd1^{\Delta\beta}$ and control (ctrl) islets at one and three weeks following TM treatment (H and I) or for $Lsd1^{\Delta\beta}$ and $Lsd1^{KI/KI\beta}$ (J) compared to respective control islets three weeks following TM treatment. Genes differentially expressed ($P < 0.01$ by Cuffdiff) in $Lsd1^{\Delta\beta}$ islets or $Lsd1^{KI/KI\beta}$ islets compared to respective control islets for either comparison and annotated to the shown functional categories (from Figure 5E) are plotted. $Lsd1^{\Delta\beta}$ week three: $n = 5$ biological replicates of islets pooled from separate mice per group, $Lsd1^{KI/KI\beta}$ and respective controls: $n = 4$ biological replicates, all other groups: $n = 3$ biological replicates.
(K) H3K4me1 ChIP-seq genome browser tracks for genes shown in Figure 5G in control and $Lsd1^{\Delta\beta}$ islets one week following TM treatment.
$^{*}P < 0.05$, $^{***}P < 0.001$ by Wilcoxon rank-sum test. NS, not significant.

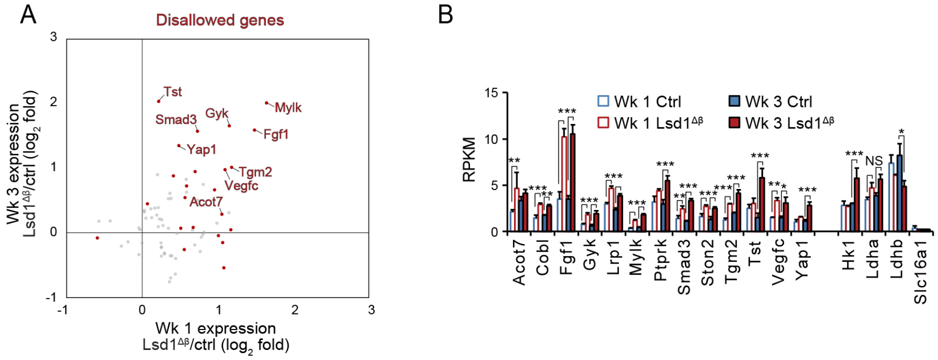

Supplemental Figure 6. Expression of disallowed genes in islets from Lsd1$^{\Delta\beta}$ mice.
(A) Log_2 fold changes in mRNA levels between Lsd1$^{\Delta\beta}$ and control (ctrl) islets at one and three weeks following TM treatment for islet-disallowed genes (3, 4) and β-cell-disallowed genes (5). Genes differentially expressed in at least one time point (*P* < 0.01 by Cuffdiff) are shown in red while genes unaffected in Lsd1$^{\Delta\beta}$ islets are shown in grey.
(B) Bar graph of mRNA levels for select disallowed genes regulated by Lsd1 (left) and for β-cell-repressed genes involved in glycolysis and lactate production (right) in Lsd1$^{\Delta\beta}$ and control (ctrl) islets at one and three weeks following TM treatment. Data shown as mean ± S.E.M. **P* < 0.05, ***P* < 0.01, ****P* < 0.001 by Cuffdiff.

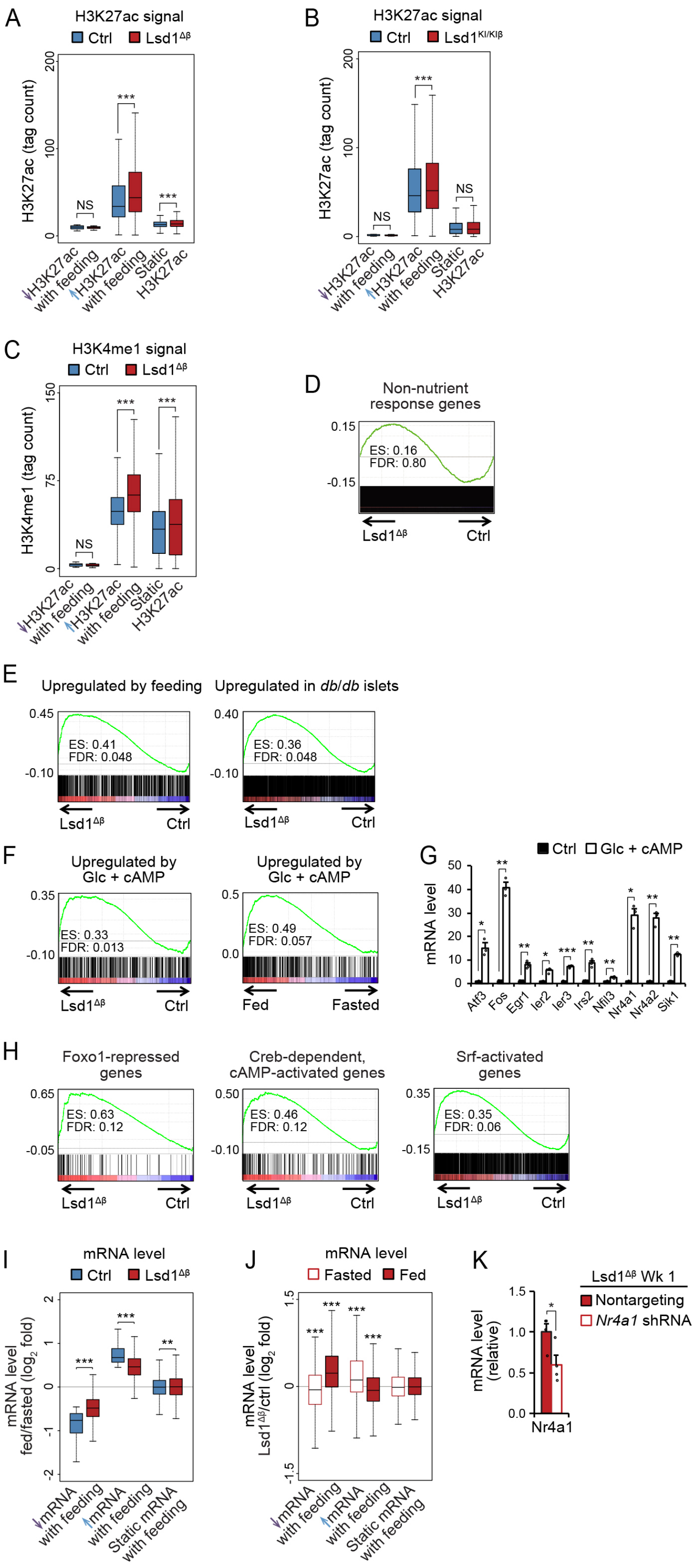

Supplemental Figure 7. *Lsd1* inactivation dysregulates nutrient response genes.

(A-C) ChIP-seq signal for H3K27ac (A, B) and H3K4me1 (C) at the indicated classes of H3K27ac peaks in control and Lsd1$^{\Delta\beta}$ islets one week following TM treatment (A and C) and in control and Lsd1$^{KI/KI\beta}$ islets three weeks following TM treatment (B). Boxplot whiskers span data points within the interquartile range x 1.5. ***P < 0.001 by Wilcoxon rank-sum test.
(D) Gene set enrichment analysis of non-nutrient response genes (for comparison with Figure 5H) against mRNA-seq data from Lsd1$^{\Delta\beta}$ and control islets one week following TM treatment. n = 3 biological replicates of islets pooled from separate mice per group.
(E) Gene set enrichment analysis of genes upregulated by feeding in islets (left, P < 0.01 by Cuffdiff) and in *db/db* compared to control (*db/+*) islets (right, P < 0.01 by Cuffdiff) against mRNA-seq data from Lsd1$^{\Delta\beta}$ and control islets one week following TM treatment.
(F) Gene set enrichment analysis of genes upregulated by glucose (Glc) and cAMP co-treatment in Min6 insulinoma cells (6) against mRNA-seq data from Lsd1$^{\Delta\beta}$ and control islets one week following TM treatment (left) or against islet mRNA-seq data from fed and fasted mice (right).
(G) qPCR of select nutrient-dependent signaling pathway genes in primary mouse islets treated with 11 mM glucose and 0.5 mM cpt-cAMP for 1 hour. n = 3 pools of 50 islets each per group. Data are shown as mean ± S.E.M. *P < 0.05, **P < 0.01, ***P < 0.001 by unpaired two-tailed t-test with Welch's correction for unequal variance as necessary.
(H) Gene set enrichment analysis for genes upregulated following β-cell *Foxo1* deletion (7) (left), mouse orthologs of genes upregulated by the cAMP-raising agent forskolin in control INS1 β-cells but not in β-cells expressing dominant-negative Creb (8) (middle), and genes upregulated following lentiviral Srf overexpression in primary islets (9) (right) against mRNA-seq data from Lsd1$^{\Delta\beta}$ and control islets one week following tamoxifen treatment.
(I and **J)** Log_2 fold changes in mRNA levels in islets during feeding and fasting in the indicated genotypes of mice (I) or in Lsd1$^{\Delta\beta}$ compared to control islets for the indicated feeding states (J) for genes classified based on their regulation by feeding in control (ctrl) islets (P < 0.01 by Cuffdiff comparing fed and fasted ctrl islets). Islets were isolated from ctrl and Lsd1$^{\Delta\beta}$ mice that were feeding-entrained and then fed or fasted as in Figure 3H one week following TM treatment. n = 2 biological replicates of islets pooled from separate mice per group. **P < 0.01, ***P < 0.001 by Wilcoxon rank-sum test for comparisons indicated with brackets (I) or between indicated classes and static mRNAs with feeding in the same state (J).
(K) qPCR for *Nr4a1* in Lsd1$^{\Delta\beta}$ islets transduced with the indicated lentiviral shRNAs. Islets were dissociated and transduced with lentiviral shRNAs 3 days following TM treatment, then RNA was isolated from islets 72 hours following transduction to confirm knockdown. n = 4 pools of reaggregated islets. *P < 0.05 by unpaired two-tailed t-test with Welch's correction for unequal variance.

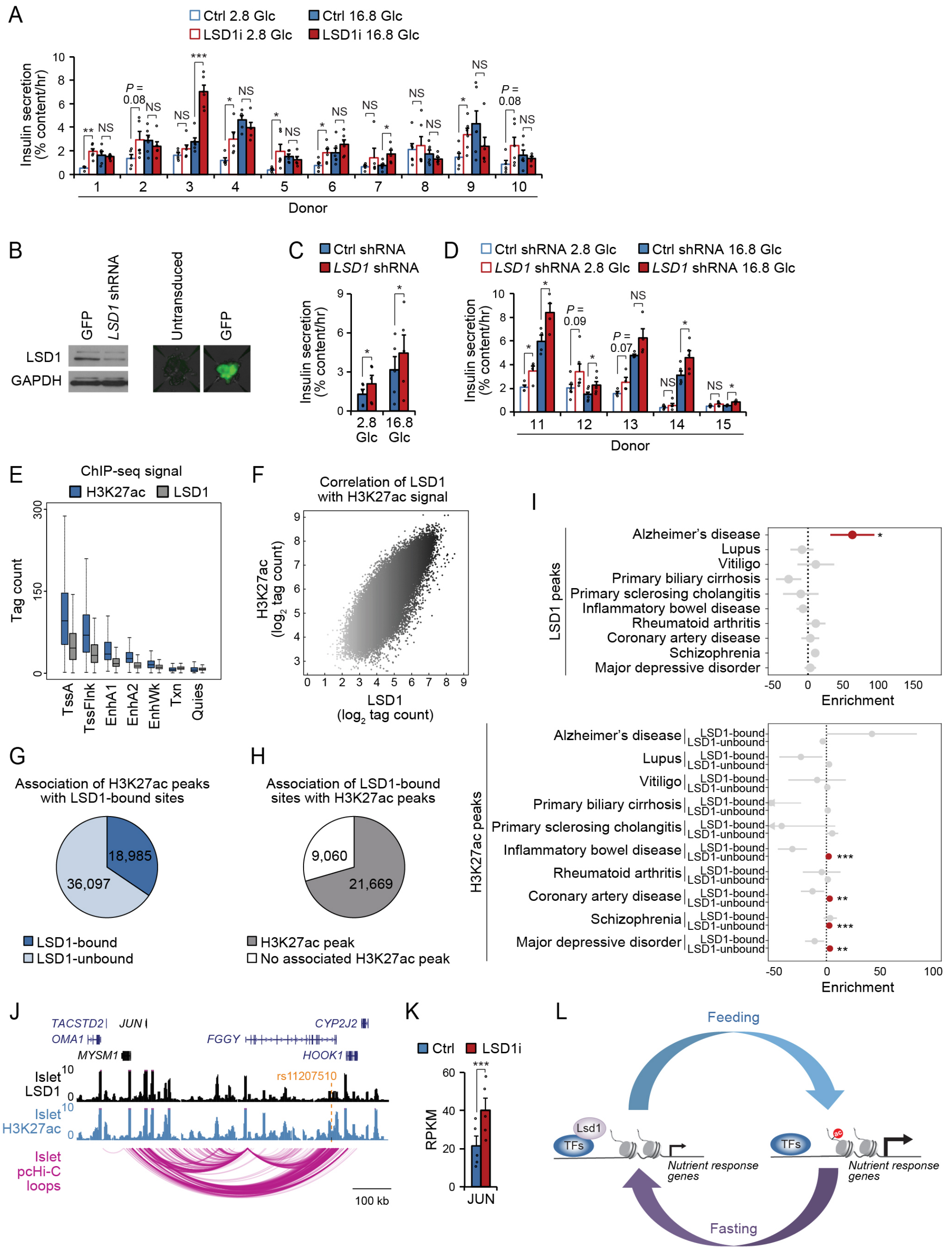

Supplemental Figure 8. LSD1 associates with active chromatin and regulates insulin secretion in human islets.

(A) Static insulin secretion assays for each human islet preparation stimulated with the indicated glucose (Glc) concentrations (in mM) following 24-hour treatment with the LSD1 inhibitor (LSD1i) TCP or vehicle control (ctrl). Donor 1 2.8 mM Glc + vehicle and 16.8 mM Glc + vehicle, Donor 2 2.8 mM Glc + vehicle, Donor 3 2.8 mM Glc + vehicle, Donor 4 all groups, donor 5 16.8 mM Glc + LSD1i, donor 7 2.8 mM Glc + LSD1i and 16.8 mM Glc + LSD1i, donor 10 2.8 mM Glc + vehicle: *n* = 5 pools of 10 islets each, all other groups: *n* = 6 pools of islets. Data are shown as mean ±

S.E.M. *$P < 0.05$, **$P < 0.01$, ***$P < 0.001$ by unpaired two-tailed t-test with Welch's correction for unequal variance as necessary. NS, not significant.
(B) Western blot for the indicated proteins (left) and GFP fluorescence overlaid with the transmitted light channel (right) in dissociated and reaggregated human islets transduced with *LSD1* shRNA lentivirus or GFP-expressing control lentivirus.
(C and **D)** Static insulin secretion assays for sorted and reaggregated human β-cells stimulated with the indicated glucose (Glc) concentrations (in mM) following transduction with *LSD1* shRNA or non-targeting (ctrl) shRNA lentiviruses shown in aggregate (C, $n = 5$ donors) or as individual donors (D). Donors 11, 13, and 15: $n = 4$ pools of 5,000 reaggregated β-cells each, donor 12: $n = 6$ pools of reaggregated β-cells, donor 14: $n = 5$ pools of reaggregated β-cells. Data are shown as mean ± S.E.M. *$P < 0.05$ by paired (C) or unpaired (D) two-tailed t-test with Welch's correction for unequal variance as necessary. NS, not significant.
(E) H3K27ac and LSD1 ChIP-seq signals at the indicated ChromHMM states in human islets. Data from independent ChIP-seq experiments from four donors (H3K27ac) or 2 donors (LSD1) were merged for analysis. Boxplot whiskers span data points within the interquartile range x 1.5.
(F) Scatterplot of ChIP-seq signals for H3K27ac and LSD1 in human islets.
(G and **H)** Numbers of H3K27ac peaks associated with an LSD1 peak ± 1 kb (G) and vice versa (H).
(I) GWAS enrichment (%h^2/%SNPs) of non-metabolic traits at LSD1 ChIP-seq peaks (top) and at H3K27ac ChIP-seq peaks associated with an LSD1 peak ± 1 kb or not associated with an LSD1 peak (bottom) in human islets using LD-score regression. Data shown represent enrichment estimate and standard error, and significant estimates are highlighted in red. *$P < 0.05$, **$P < 0.01$, ***$P < 0.001$ (Bonferroni-corrected) for enrichment by LD-score regression. Data from independent ChIP-seq experiments from four donors (H3K27ac) or two donors (LSD1) were merged for analysis.
(J) LSD1 and H3K27ac ChIP-seq and promoter capture Hi-C (pcHi-C)(10) genome browser tracks showing interaction between the LSD1-bound site containing T2D-associated variant rs11207510 (highlighted in orange) and the *JUN* promoter in human islets.
(K) Bar graph of *JUN* mRNA level in human islets treated with LSD1i as in Figure 6A. Data shown as mean ± S.E.M. ***$P < 0.001$ by Cuffdiff.
(L) Working model for Lsd1-mediated regulation of the β-cell epigenome during feeding and fasting.

Supplemental Tables

Supplemental Table 1. mRNA-seq and ChIP-seq replicate correlations.
Pearson correlations for log-transformed RPKM data across all genes (mRNA-seq) or Pearson correlations genomewide (ChIP-seq) between all replicates for each experiment. For ChIP-seq experiments with *n* = 2, pairwise comparisons are given.

Islets from fed and fasted mice mRNA-seq

Fast A	Fast B	Fast C	Fed A	Fed B	Fed C	
1.000	0.976	0.966	0.966	0.971	0.957	Fast A
	1.000	0.965	0.972	0.970	0.957	Fast B
		1.000	0.968	0.971	0.962	Fast C
			1.000	0.973	0.963	Fed A
				1.000	0.969	Fed B
					1.000	Fed C

Islets from *db/db* and *db/+* mice mRNA-seq

db/db A	*db/db* B	*db/db* C	*db/+* A	*db/+* B	*db/+* C	
1.000	0.975	0.979	0.955	0.959	0.965	*db/db* A
	1.000	0.981	0.959	0.962	0.968	*db/db* B
		1.000	0.960	0.964	0.968	*db/db* C
			1.000	0.971	0.975	*db/+* A
				1.000	0.973	*db/+* B
					1.000	*db/+* C

Islets from Lsd1$^{\Delta\beta}$ and control (ctrl) mice mRNA-seq

Wk1 Ctrl A	Wk1 Ctrl B	Wk1 Ctrl C	Wk1 Lsd1$^{\Delta\beta}$ A	Wk1 Lsd1$^{\Delta\beta}$ B	Wk1 Lsd1$^{\Delta\beta}$ C	Wk3 Ctrl A	Wk3 Ctrl B	Wk3 Ctrl C	Wk3 Lsd1$^{\Delta\beta}$ A	Wk3 Lsd1$^{\Delta\beta}$ B	Wk3 Lsd1$^{\Delta\beta}$ C	Wk3 Lsd1$^{\Delta\beta}$ D	Wk3 Lsd1$^{\Delta\beta}$ E	
1.000	0.973	0.974	0.965	0.964	0.962	0.962	0.965	0.966	0.957	0.961	0.959	0.960	0.952	Wk1 Ctrl A
	1.000	0.975	0.964	0.969	0.963	0.964	0.964	0.965	0.957	0.959	0.962	0.961	0.960	Wk1 Ctrl B
		1.000	0.965	0.968	0.966	0.966	0.968	0.965	0.964	0.963	0.968	0.961	0.957	Wk1 Ctrl C
			1.000	0.973	0.971	0.960	0.964	0.964	0.964	0.963	0.966	0.969	0.963	Wk1 Lsd1$^{\Delta\beta}$ A
				1.000	0.975	0.964	0.966	0.965	0.969	0.964	0.970	0.973	0.969	Wk1 Lsd1$^{\Delta\beta}$ B
					1.000	0.962	0.966	0.963	0.970	0.965	0.971	0.972	0.966	Wk1 Lsd1$^{\Delta\beta}$ C
						1.000	0.960	0.963	0.959	0.953	0.964	0.963	0.956	Wk3 Ctrl A
							1.000	0.965	0.966	0.958	0.962	0.964	0.962	Wk3 Ctrl B
								1.000	0.960	0.961	0.963	0.960	0.956	Wk3 Ctrl C
									1.000	0.967	0.970	0.965	0.963	Wk3 Lsd1$^{\Delta\beta}$ A
										1.000	0.971	0.959	0.954	Wk3 Lsd1$^{\Delta\beta}$ B
											1.000	0.965	0.962	Wk3 Lsd1$^{\Delta\beta}$ C
												1.000	0.969	Wk3 Lsd1$^{\Delta\beta}$ D
													1.000	Wk3 Lsd1$^{\Delta\beta}$ E

Islets from Lsd1$^{KI/KI\beta}$ and control (ctrl) mice mRNA-seq

Ctrl A	Ctrl B	Ctrl C	Ctrl D	Lsd1$^{KI/KI\beta}$ A	Lsd1$^{KI/KI\beta}$ B	Lsd1$^{KI/KI\beta}$ C	Lsd1$^{KI/KI\beta}$ D	
1.000	0.995	0.994	0.994	0.988	0.989	0.989	0.989	Ctrl A
	1.000	0.995	0.995	0.987	0.989	0.988	0.987	Ctrl B
		1.000	0.995	0.990	0.990	0.989	0.990	Ctrl C
			1.000	0.989	0.989	0.988	0.990	Ctrl D
				1.000	0.995	0.995	0.994	Lsd1$^{KI/KI\beta}$ A
					1.000	0.995	0.995	Lsd1$^{KI/KI\beta}$ B
						1.000	0.994	Lsd1$^{KI/KI\beta}$ C
							1.000	Lsd1$^{KI/KI\beta}$ D

Islets from fed and fasted Lsd1$^{\Delta\beta}$ and control (ctrl) mice mRNA-seq

Ctrl Fast A	Ctrl Fast B	Ctrl Fed A	Ctrl Fed B	Lsd1$^{\Delta\beta}$ Fast A	Lsd1$^{\Delta\beta}$ Fast B	Lsd1$^{\Delta\beta}$ Fed A	Lsd1$^{\Delta\beta}$ Fed B	
1.000	0.995	0.990	0.993	0.993	0.989	0.988	0.990	Ctrl Fast A
	1.000	0.993	0.992	0.992	0.990	0.989	0.991	Ctrl Fast B
		1.000	0.995	0.987	0.985	0.992	0.990	Ctrl Fed A
			1.000	0.992	0.987	0.993	0.993	Ctrl Fed B
				1.000	0.993	0.993	0.995	Lsd1$^{\Delta\beta}$ Fast A
					1.000	0.988	0.991	Lsd1$^{\Delta\beta}$ Fast B
						1.000	0.996	Lsd1$^{\Delta\beta}$ Fed A
							1.000	Lsd1$^{\Delta\beta}$ Fed B

1.000	0.960	0.970	0.951	0.962	0.943	0.956	0.944	0.959	LSD1i A
	1.000	0.957	0.967	0.963	0.956	0.956	0.959	0.960	Ctrl B
		1.000	0.952	0.965	0.943	0.958	0.945	0.959	LSD1i B
			1.000	0.974	0.969	0.968	0.972	0.970	Ctrl C
				1.000	0.964	0.974	0.965	0.976	LSD1i C
					1.000	0.972	0.973	0.969	Ctrl D
						1.000	0.970	0.978	LSD1i D
							1.000	0.975	Ctrl E
								1.000	LSD1i E

Histone modification ChIP-seq

Sample	H3K27ac	H3K4me1
Wk1 Ctrl	0.915	0.973
Wk1 Lsd1$^{\Delta\beta}$	0.932	0.943
Wk3 Ctrl	0.864	0.968
Wk3 Lsd1$^{\Delta\beta}$	0.914	0.977
Fasted	0.866*	0.972
Fed	0.876*	0.973
db/+	0.925	0.956
db/db	0.838	0.974

*Original H3K27ac ChIP-seq for initial peak calling

Islets from fed and fasted mice H3K27ac ChIP-seq

Fast A	Fast B	Fast C	Fed A	Fed B	Fed C	
1.000	0.989	0.974	0.975	0.959	0.942	Fast A
	1.000	0.987	0.988	0.978	0.965	Fast B
		1.000	0.994	0.990	0.985	Fast C
			1.000	0.993	0.987	Fed A
				1.000	0.995	Fed B
					1.000	Fed C

Islets from Lsd1$^{KI/KI\beta}$ H3K27ac ChIP-seq

Ctrl A	Ctrl B	Lsd1$^{KI/KI\beta}$ A	Lsd1$^{KI/KI\beta}$ B	Lsd1$^{KI/KI\beta}$ C	
1.000	0.992	0.981	0.987	0.975	Ctrl A
	1.000	0.982	0.982	0.968	Ctrl B
		1.000	0.990	0.989	Lsd1$^{KI/KI\beta}$ A
			1.000	0.990	Lsd1$^{KI/KI\beta}$ B
				1.000	Lsd1$^{KI/KI\beta}$ C

Other ChIP-seq

Sample	Lsd1	Srf
Mouse Islets	0.892	
Human Islets	0.778	
Min6 Cells		0.724

Islets from fed and fasted mice Lsd1 ChIP-seq

Fast A	Fast B	Fed A	Fed B	
1.000	0.866	0.836	0.845	Fast A
	1.000	0.841	0.892	Fast B
		1.000	0.795	Fed A
			1.000	Fed B

Supplemental Table 2. Differentially expressed genes and enriched gene ontologies.
(**A-D**) Differentially expressed genes (P < 0.01 by Cuffdiff) in islets from fed compared to fasted mice (A), in islets from *db/db* compared to *db/+* mice (B), in Lsd1$^{\Delta\beta}$ or Lsd1$^{KI/KI\beta}$ compared to control islets (C), and in LSD1i-treated compared to control human islets (D).
(**E-G**) Gene ontology terms for genes differentially expressed only at one week (E), only at three weeks (F), and at both time points (G) following *Lsd1* deletion.
(supplied as Excel file: TableS2.xlsx)

Supplemental Table 3. ChIP-seq peak coordinates.
(**A**) H3K27ac peaks classified by changes in response to feeding (P < 0.01 by DEseq2).
(**B**) Lsd1 peaks in islets from ad libitum-fed mice.
(**C**) H3K27ac peaks classified by changes in response to feeding and association (or lack thereof) with an Lsd1 peak ± 1 kb.
(**D** and **E**) H3K27ac peaks (D) and H3K4me1 peaks (E) classified by changes in Lsd1$^{\Delta\beta}$ compared to control islets (P < 0.01 by DEseq2).
(**F** and **G**) LSD1 peaks (F) and H3K27ac peaks (G) in human islets.
See Methods for details about peak calling and classification.
(supplied as Excel file: TableS3.xlsx)

Supplemental Table 4. Enrichment of transcription factor (TF) motifs in H3K27ac peak classes.
(**A**) TF motifs enriched in H3K27ac peaks gaining acetylation with feeding relative to static H3K27ac peaks.
(**B**) TF motifs enriched in peaks gaining acetylation with feeding and associated with an Lsd1 peak ± 1 kb relative to H3K27ac peaks gaining acetylation with feeding and not associated with an Lsd1 peak.
(**C**) TF motifs enriched in peaks gaining acetylation with feeding and associated with an Lsd1 peak ± 1 kb relative to static H3K27ac peaks associated with an Lsd1 peak ± 1 kb.
See Methods for details about peak calling and classification.
(supplied as Excel file: TableS4.xlsx)

Supplemental Table 5. Promoters interacting with sites containing T2D-associated variants.
(**A** and **B**) Genes whose promoters interact with an LSD1-bound (A) or LSD1-unbound (B) site containing a type 2 diabetes (T2D)-associated variant from promoter capture Hi-C data.
(supplied as Excel file: TableS5.xlsx)

Supplemental Table 6. Human islet donor information.
Donor information, isolation center, and data generated for each human islet prep. All tissue was obtained through the Integrated Islet Distribution Program. All donors were nondiabetic.

Human islet donor information

RRID:	SAMN 08775076	SAMN 08775042	SAMN 08930500	SAMN 08775026	SAMN 08774953	SAMN 08930577	SAMN 08773781	SAMN 08773777	SAMN 08769126	SAMN 08769081
Age	59	48	35	47	29	50	42	49	35	52
Sex	M	F	F	F	M	M	F	F	M	M
BMI	21.5	31	34.7	29.9	32.6	31.7	23.1	31.6	27.4	22.1
HbA1c	ND	ND	ND	ND	ND	ND	5.4%	5.2%	5.4%	5.6%
Islet isolation center	Scharp-Lacy	Scharp-Lacy	Illinois	Miami	Miami	Illinois	Penn	Penn	City of Hope	Scharp-Lacy
Cause of death	Anoxia	CV/stroke	CV/stroke	CV/stroke	Head trauma	Head trauma	CV/stroke	CV/stroke	CV/stroke	CV/stroke
Use in study	LSDi:GSIS	LSDi:GSIS	LSDi:GSIS mRNA-seq	LSDi:GSIS	LSDi:GSIS mRNA-seq	LSDi:GSIS	LSDi:GSIS	LSDi:GSIS mRNA-seq	LSDi:GSIS	LSDi:GSIS
Alias	Donor 1	Donor 2	Donor 3	Donor 4	Donor 5	Donor 6	Donor 7	Donor 8	Donor 9	Donor 10

Human islet donor information (continued)

RRID:	SAMN 10737781	SAMN 11476721	SAMN 11522709	SAMN 12070499	SAMN 12123357	SAMN 08784602	SAMN 08769028	SAMN 08774194	SAMN 08774189
Age	66	50	51	60	47	24	63	68	53
Sex	M	M	M	M	M	F	F	M	M
BMI	27.2	32.8	26.7	34.8	28.5	34.9	20	26.7	21.8
HbA1c	4.7%	6.0%	5.7%	4.0%	5.8%	ND	5.0%	5.3%	5.5%
Islet isolation center	Scharp-Lacy	Scharp-Lacy	City of Hope	Miami	Scharp-Lacy	Scharp-Lacy	Miami	Scharp-Lacy	City of Hope
Cause of death	CV/stroke	Anoxia	Anoxia	Head trauma	Head trauma	Head trauma	CV/stroke	Head trauma	Head trauma
Use in study	shRNA: GSIS	shRNA: GSIS	shRNA: GSIS	shRNA: GSIS	shRNA: GSIS	LSD1 ChIP-seq	LSD1 ChIP-seq	LSDi: mRNA-seq	LSDi: mRNA-seq
Alias	Donor 11	Donor 12	Donor 13	Donor 14	Donor 15	N/A	N/A	N/A	N/A

ND, not determined; CV, cardiovascular; LSDi, LSD1 inhibition with tranylcypromine; Sharp-Lacy, The Sharp-Lacy Research Institute; Illinois, University of Illinois; Miami, University of Miami; Penn, University of Pennsylvania; City of Hope, Southern California Islet Cell Resource Center at City of Hope.

Supplemental Table 7. shRNA mature antisense sequences.

Experiment	Group	Sequence (source if applicable)
LSD1 (human β-cells)	Nontargeting	CCTAAGGTTAAGTCGCCCTCG
LSD1 (human β-cells)	LSD1 #1	TATTCAGTTTAATGTCTAGGC
LSD1 (human β-cells)	LSD1 #2	TAGTGCCAACAGTATTGGAGC
LSD1 (human β-cells)	LSD1 #3	TAAGGTGCTTCTAATTGTTGG
LSD1 (human β-cells)	LSD1 #4	ATGACTAAGGTAAGATGTAGC
Nr4a1 (mouse islets)	Nontargeting	CTATAGGCTTAAAGGCACATG
Nr4a1 (mouse islets)	Nr4a1 #1	GATAACGTCCAGGGAACCAGA
Nr4a1 (mouse islets)	Nr4a1 #2	CTATAGGCTTAAAGGCACATG
Nr4a1 (mouse islets)	Nr4a1 #3	TAAAGATCTTGTCCACAATAGT
Nr4a1 (mouse islets)	Nr4a1 #4	TTTCTGTACTGTGCGCTTG (11)

Supplemental Table 8. RT-qPCR primer sequences.

mRNA	F Primer	R Primer
Atf3	CCCCTGGAGATGTCAGTCAC	CGGTGCAGGTTGAGCATGT
Egr1	GCGAACAACCCTATGAGCAC	GAGGATGAAGAGGTCGGAGG
Fos	CGGGTTTCAACGCCGACTA	TTGGCACTAGAGACGGACAGA
Gapdh	CATGTTCCAGTATGACTCCACTC	GGCCTCACCCCATTTGATGT
Ier2	TTTGAGCGACGGTAGTGATGC	GAGACTGGAGAAGCGCCTTTG
Ier3	CAGCCGAAGGGTGCTCTAC	AGCCATCAAAATCTGGCAGAAG
Irs2	GCTTGAAGCGGCTAAGTCTC	GACGGTGGTGGTAGAGGAAA
Nfil3	TGGAGCACACTCAGGAAAGC	ATGGACCTGCAAGAGGGGT
Nr4a1	GACTTGCTCTCTGGTTCCCT	AGAAGGCCAGGATGTTGTCA
Nr4a2	GTGAGGGCTGCAAAGGTTTC	CGGCCTTTTAAACTGTCCGT
Sik1	CCAACCTGCCTACGCTGAG	GATCTGGGCTATGGTGATGCG

References

1. Nammo T, et al. Genome-wide profiling of histone H3K27 acetylation featured fatty acid signalling in pancreatic beta cells in diet-induced obesity in mice. *Diabetologia.* 2018;61(12):2608-20.
2. Lu TT, et al. The polycomb-dependent epigenome controls beta cell dysfunction, dedifferentiation, and diabetes. *Cell Metab.* 2018;27(6):1294-308 e7.
3. Pullen TJ, et al. Identification of genes selectively disallowed in the pancreatic islet. *Islets.* 2010;2(2):89-95.
4. Thorrez L, et al. Tissue-specific disallowance of housekeeping genes: the other face of cell differentiation. *Genome Res.* 2011;21(1):95-105.
5. Pullen TJ, et al. Analysis of purified pancreatic islet beta and alpha cell transcriptomes reveals 11beta-hydroxysteroid dehydrogenase (Hsd11b1) as a novel disallowed gene. *Front Genet.* 2017;8:41.
6. Glauser DA, et al. Transcriptional response of pancreatic beta cells to metabolic stimulation: large scale identification of immediate-early and secondary response genes. *BMC Mol Biol.* 2007;8:54.
7. Kuo T, et al. Induction of alpha cell-restricted Gc in dedifferentiating beta cells contributes to stress-induced beta-cell dysfunction. *JCI Insight.* 2019;5.
8. Blanchet E, et al. Feedback inhibition of CREB signaling promotes beta cell dysfunction in insulin resistance. *Cell Rep.* 2015;10(7):1149-57.
9. Zeng C, et al. Pseudotemporal ordering of single cells reveals metabolic control of postnatal beta cell proliferation. *Cell Metab.* 2017;25(5):1160-75 e11.
10. Miguel-Escalada I, et al. Human pancreatic islet three-dimensional chromatin architecture provides insights into the genetics of type 2 diabetes. *Nat Genet.* 2019;51(7):1137-48.
11. Zhang Y, et al. NR4A1 knockdown suppresses seizure activity by regulating surface expression of NR2B. *Sci Rep.* 2016;6:37713.
